# Identification of Potential Abnormal Methylation-Modified Genes in Coronary Artery Ectasia

**DOI:** 10.1155/2023/4969605

**Published:** 2023-08-26

**Authors:** Xiuchun Yang, Yijun Zong, Zhentian Zhang, Yan Zhao, Xueying Gao, Jie Zhang, Qian Hou, Renyi Li, Bing Xiao

**Affiliations:** ^1^Department of Cardiology, The Second Hospital of Hebei Medical University, Shijiazhuang, China; ^2^School of Nursing, Hebei University of Chinese Medicine, Shijiazhuang, China; ^3^Department of Neurology, The Second Hospital of Hebei Medical University, Shijiazhuang, China

## Abstract

**Background:**

Coronary artery ectasia (CAE) is an easily recognized abnormality of coronary artery anatomy and morphology. However, its pathogenesis remains unclear.

**Objectives:**

This study aimed to identify abnormal methylation-modified genes in patients with CAE, which could provide a research basis for CAE.

**Methods:**

Peripheral venous blood samples from patients with CAE were collected for RNA sequencing to identify differentially expressed genes (DEGs), followed by functional enrichment. Then, the DNA methylation profile of CAE was downloaded from GSE87016 (HumanMethylation450 BeadChip data, involving 11 cases and 12 normal controls) to identify differentially methylated genes (DMGs). Finally, after taking interaction genes between DEGs and DMGs, abnormal methylation-modified genes were identified, followed by protein–protein interaction analysis and expression validation using reverse transcriptase polymerase chain reaction.

**Results:**

A total of 152 DEGs and 4318 DMGs were obtained from RNA sequencing and the GSE87016 dataset, respectively. After taking interaction genes, 9 down-regulated DEGs due to hypermethylation and 11 up-regulated DEGs due to hypomethylation were identified in CAE. A total of 10 core abnormal methylation-modified genes were identified, including six down-regulated DEGs due to hypermethylation (netrin G1, ADAM metallopeptidase domain 12, immunoglobulin superfamily member 10, sarcoglycan dela, Dickkopf WNT signaling pathway inhibitor 3, and GATA binding protein 6), and four up-regulated DEGs due to hypomethylation (adrenomedullin, ubiquitin specific peptidase 18, lymphocyte antigen 6 family member E, and MX dynamin-like GTPase 1). Some signaling pathways were identified in patients with CAE, including cell adhesion molecule, O-glycan biosynthesis, and the renin–angiotensin system.

**Conclusions:**

Abnormal methylation-modified DEGs involved in signaling pathways may be involved in CAE development.

## 1. Introduction

The cardiovascular system is essential in several key physiological processes [1]. Coronary artery ectasia (CAE), a rare disease, is defined as the dilation of the coronary vascular lumen (up to a diameter 1.5 times that of the adjacent normal coronary artery) [2, 3]. It is an inflammatory disease that develops in the background of atherosclerosis, with a prevalence of 1.2–4.9% [4, 5]. Angina pectoris is the most common clinical feature of CAE [6]. Acute coronary syndrome, dysrhythmia, and sudden cardiac death are other possible clinical situations observed in patients with CAE [7–9]. Factors associated with CAE, include infections, iatrogenic disease, cardiac lymphomas, congenital and collagen vascular disorders, sex (male), hyperlipidemia, hypertension, smoking, stroke history, and cocaine use [10–14]. However, the pathogenesis of CAE remains unclear.

Information regarding differentially expressed genes (DEGs) can be obtained from RNA sequencing. In addition, DNA methylation can inhibits gene transcription, and in the promoters and enhancers, DNA methylation is related to gene silencing [15]. Recently, there have been limited investigations on gene expression and DNA methylation in CAE. Therefore, our study aimed to identify DEGs and differentially methylated genes (DMGs) in CAE using RNA sequencing and integration analysis, followed by functional analysis of abnormal methylation-modified genes. This study may lay the molecular foundation for the development of precision medicine for patients with CAE.

## 2. Methods

### 2.1. Patients and Samples

This study was conducted with five patients with CAE and five healthy controls. The clinical information of these individuals is listed in Table 1. The inclusion criteria for patients with CAE were as follows: patients diagnosed with CAE with localized or diffuse dilatation of the coronary artery wall, with the diameter of the diseased lumen exceeding 1.5 times or more of the adjacent normal segment and without coronary artery stenosis (<50%). The exclusion criteria for patients with CAE were as follows: (1) patients with a history of old myocardial infarction; (2) patients previously treated with coronary intervention or coronary artery bypass grafting; (3) patients with congenital heart disease or other cardiac diseases (such as dilated cardiomyopathy, hypertrophic cardiomyopathy, valvular heart disease, severe arrhythmia, pulmonary heart disease, pericarditis, pericardial effusion, myocarditis, and so on); (4) patients with severe hepatic and renal insufficiency; (5) patients with malignancies, immune diseases, Kawasaki disease, and connective tissue diseases (such as systemic lupus erythematosus, aortitis, and so on); (6) patients with a recent history of trauma or surgery; (7) patients were pregnant women; and (8) patients have a history of anemia or have previously received blood transfusions or treatment for anemia (such as iron supplements, folic acid, erythropoietin, and so on). Peripheral venous blood samples were obtained from these individuals for RNA sequencing. Written informed consent was obtained from these individuals. This study was approved by the Ethics Committee of the Second Hospital of Hebei Medical University (2021-R118).

### 2.2. RNA Sequencing

Total RNA was extracted from peripheral venous blood samples and purified using the TRIzol reagent and the RNeasy Mini Kit. An Agilent 2100 Bioanalyzer was used to determine the quality of the sequencing library. The Illumina HiSeq platform was applied to perform RNA sequencing. Original image data was converted into sequence data (FASTQ format) through base calling. The Trimmomatic software was used to preprocess the original data by using default parameters. After quality control, the high-quality sequence was compared with the reference genome (GRCh38) using HISAT2.

### 2.3. Data Preprocessing

Reference gene sequences and annotation files were used as databases to identify the expression abundance of each gene in each sample through sequence similarity comparisons. The HTSeq-count software was used to determine the number of reads aligned with the genes. After the counts were obtained by comparison, those genes with zero reads were removed. The fragments per kilobase of transcript per million fragments mapped method was used for the calculation of gene expression amount. Pearson's correlation analysis was used to explore the degree of similarity between samples. In addition, principal component analysis (PCA) was performed using the “prcomp” function in the R package. All settings were default parameters. PCA reduces the large amount of gene expression information contained in samples to a few unrelated principal components for comparison between samples.

### 2.4. Identification of DEGs in RNA Sequencing

First, the genes were filtered by counts. Only genes with counts >2 were used for further analysis. DESeq2 was used to standardize the number of counts for each gene and calculate the fold change (FC). A negative binomial test was utilized to conduct the test of difference significance. Finally, DEGs were screened under the threshold value of *p* < 0.05 and |log_2_FC| >1. Log_2_FC > 1 and log_2_FC < 1 represent up-regulation and down-regulation, respectively.

### 2.5. Functional Enrichment of DEGs

Gene Ontology (GO) and Kyoto Encyclopedia of Genes and Genomes (KEGG) analyses were performed for all DEGs in RNA sequencing by using the Database for Annotation, Visualization, and Integrated Discovery. Significantly enriched GO and KEGG terms were screened using the criterion of *p* < 0.05. Additionally, the Gene Set Enrichment Analysis (GSEA) software was used for the functional analysis of the expression matrix of all genes in RNA sequencing. The enrichment score in GSEA reflects the degree to which a gene set S is overrepresented at the extremes (top or bottom) of the entire ranked list L [16]. The “c2.Cp.Kegg.V7.4.Symbols.GMT” was used as the reference gene set. Significantly enriched signaling pathways were identified using the selection criterion of *p* < 0.05.

### 2.6. Identification of DMGs from Online Dataset

Keywords of “coronary artery ectasia” and “Homo sapiens” [porgn:__txid9606] were used for the database search. Studying type of datasets was “Methylation Profiling by array”. After screening, one methylation data set, GSE87016 (HumanMethylation450 BeadChip data, involving 11 cases and 12 normal controls), was obtained. Dataset GSE87016 was downloaded from the Gene Expression Omnibus database. In the CHAMP package, champ.norm was used to standardize the data. “Champ.DMP” was used for differential methylation analysis of genes under the selection criterion of *p* < 0.05.

### 2.7. Identification of Abnormal Methylation-Modified DEGs

Intersection genes between down-regulated genes in RNA sequencing and hypermethylated genes in the GSE42057 dataset were regarded as down-regulated DEGs due to hypermethylation. In addition, intersection genes between up-regulated genes in RNA sequencing and hypomethylated genes in the GSE42057 dataset were considered as up-regulated DEGs due to hypomethylation.

### 2.8. Protein–Protein Interaction Analysis of Abnormal Methylation-Modified DEGs

To explore the interactions between proteins encoded by abnormal methylation-modified DEGs, a protein–protein interaction (PPI) network was constructed using the online STRING database. The PPI network parameters used in STRING were combined_score >0.15. The result obtained from the STRING database was imported into the Cytoscape software. The CytoHubba plug-in was used to filter core genes because it implements 11 node ranking methods to evaluate the importance of nodes in a biological network. In this study, three algorithms were adopted, including Maximal Clique Centrality (MCC), Degree, and Edge Percolated Component (EPC). Chin et al. provided specific explanations for MCC, Degree, and EPC [17]. The core genes were screened after the intersection of the first 10 genes of each algorithm.

### 2.9. Expression Validation of Abnormal Methylation-Modified DEGs by Reverse Transcriptase Polymerase Chain Reaction

Reverse transcriptase polymerase chain reaction (RT-PCR) was used to validate the expression of abnormal methylation-modified DEGs in blood samples from patients with CAE and healthy controls. The inclusion and exclusion criteria for patients with CAE and healthy controls were the same as those for RNA sequencing. There were no significant differences in age, sex, and body mass index between the CAE and healthy control groups. Based on the above inclusion and exclusion criteria, five patients with CAE and five healthy controls were enrolled in the present study. The clinical information of these individuals is listed in Table 2. The blood samples of these individuals were collected for RT-PCR. Glyceraldehyde-3-phosphate dehydrogenase (GAPDH) and beta-actin (ACTB) were used as internal references. 2^−△△ct^ represents the relative expression of the gene. 2^−△△ct^ > 1 and 2^−△△ct^ < 1 represent up-regulation and down-regulation, respectively.

## 3. Results

### 3.1. Identification and Functional Analysis of DEGs in RNA Sequencing in Patients with CAE

**Figure 1:**
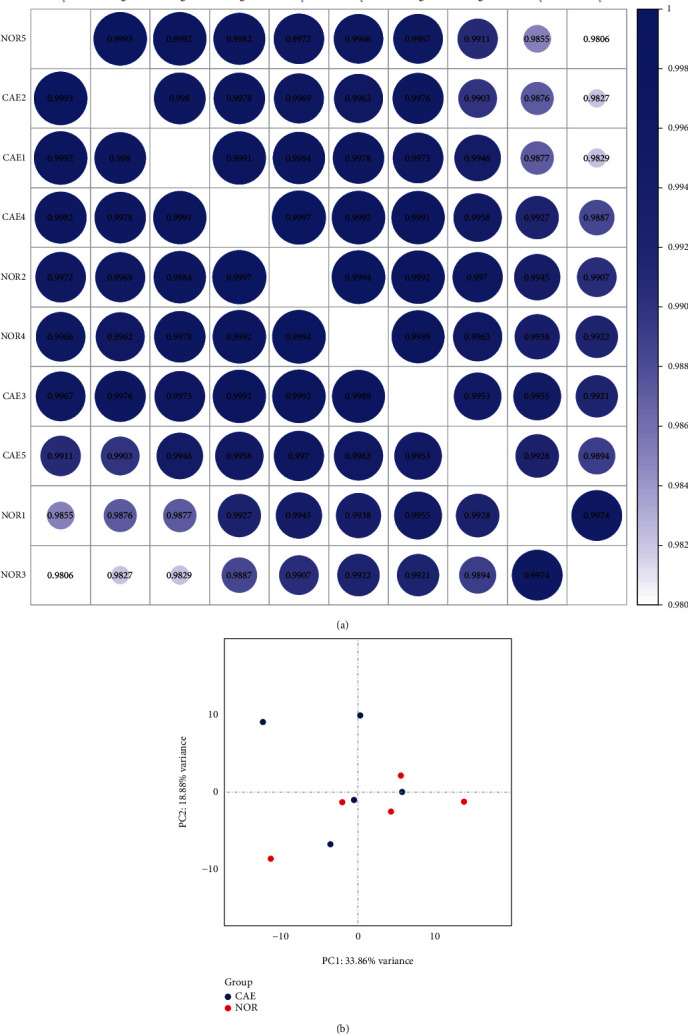
The correlation analysis between 10 (5 CAE and 5 normal controls) samples. (a) Heat map of the correlation coefficient between samples. Large scale and dark color represent a large correlation. (b) Principal component analysis. CAE: coronary artery ectasia; NOR: normal controls.

**Figure 2:**
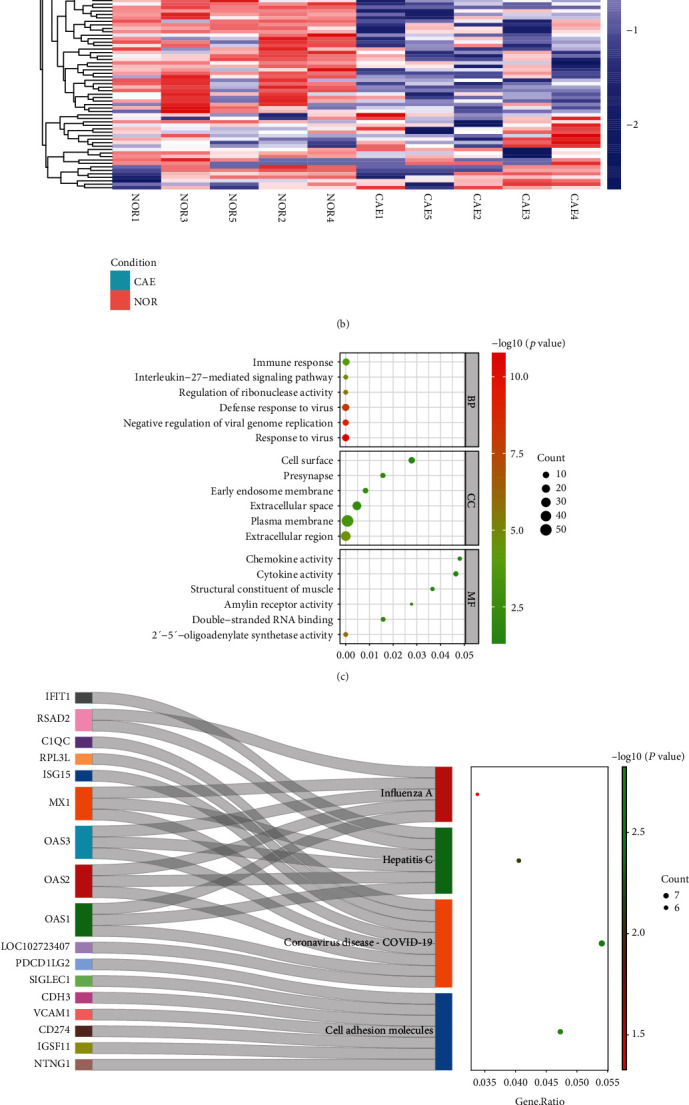
Identification and functional analysis of 152 DEGs in RNA sequencing in CAE. (a) Volcanic maps of DEGs. (b) Heat maps of DEGs. Red and blue represent up-regulated and down-regulated differentially expressed mRNAs, respectively. The darker the red color, the higher the expression level, whereas the darker the blue color, the lower the expression level. (c) GO enrichment analysis of DEGs, the *x*-axis represents *p* value. (d) KEGG enrichment analysis of DEGs, the *x*-axis represents *p* value. (e) O-glycan biosynthesis signal pathway in GSEA. (f) Fenin–angiotensin system signal pathway in GSEA. CAE: coronary artery ectasia; NOR: normal controls.

Correlation analysis (Figure 1(a)) and PCA (Figure 1(b)) showed that the similarity between the 10 samples was relatively high. This indicates that the heterogeneity between the samples was relatively small. A total of 152 DEGs were identified in patients with CAE, including 93 up-regulated and 59 down-regulated genes (Supplementary Table 1). Volcanic and heat maps of all DEGs are shown in Figures 2(a) and 2(b), respectively. Based on GO analysis, response to the virus, extracellular region, and 2′–5′-oligoadenylate synthetase activity were, respectively, the most significantly enriched biological process, cytological component, and molecular function of DEGs (Figure 2(c)). Cell adhesion molecule was the most significantly enriched signaling pathway of DEGs (Figure 2(d)). One of the DEGs, netrin G1 (NTNG1) is involved in the signaling pathway (Table 3). Additionally, two significantly down-regulated signaling pathways were identified in CAE by GSEA, including O-glycan biosynthesis (Figure 2(e)) and renin–angiotensin system (Figure 2(f)).

### 3.2. Identification of Abnormal Methylation-Modified DEGs in CAE

**Figure 3:**
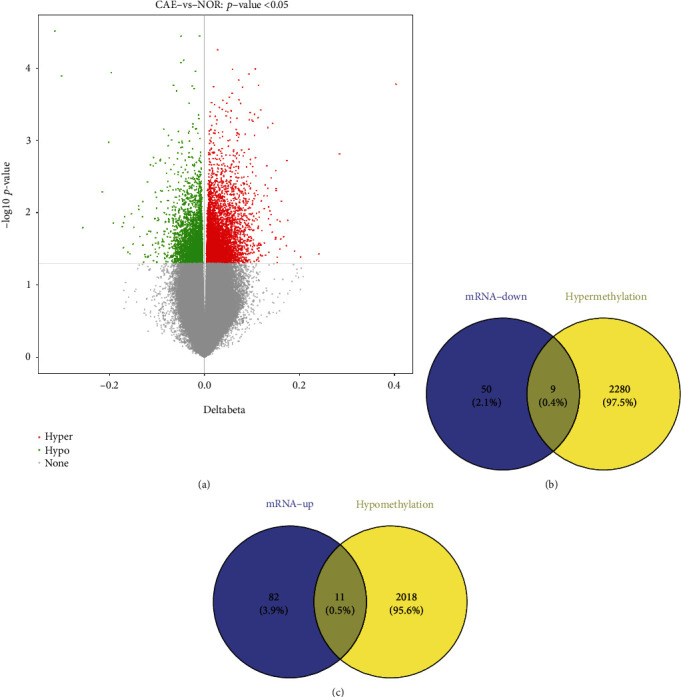
Identification of 20 abnormal methylation-modified DEGs in CAE. (a) Volcanic maps of all DMGs. (b) Venn diagram of nine down-regulated DEGs due to hypermethylation. (c) Venn diagram of 11 up-regulated DEGs due to hypomethylation.

In total, 9377 differentially methylated sites were obtained from the GSE87016 dataset in CAE. These differentially methylated sites correspond to 4318 DMGs, including 2289 hypermethylated genes and 2029 hypomethylated genes (Supplementary Table 2). The volcanic map of all DMGs is shown in Figure 3(a), respectively. After taking the intersection genes between 59 down-regulated genes and 2289 hypermethylated genes, and 93 up-regulated genes and 2029 hypomethylated genes, a total of 9 down-regulated DEGs due to hypermethylation (Figure 3(b)) and 11 up-regulated DEGs due to hypomethylation (Figure 3(c)) were identified in patients with CAE.

### 3.3. PPI Network of 20 Abnormal Methylation-Modified DEGs in CAE

**Figure 4:**
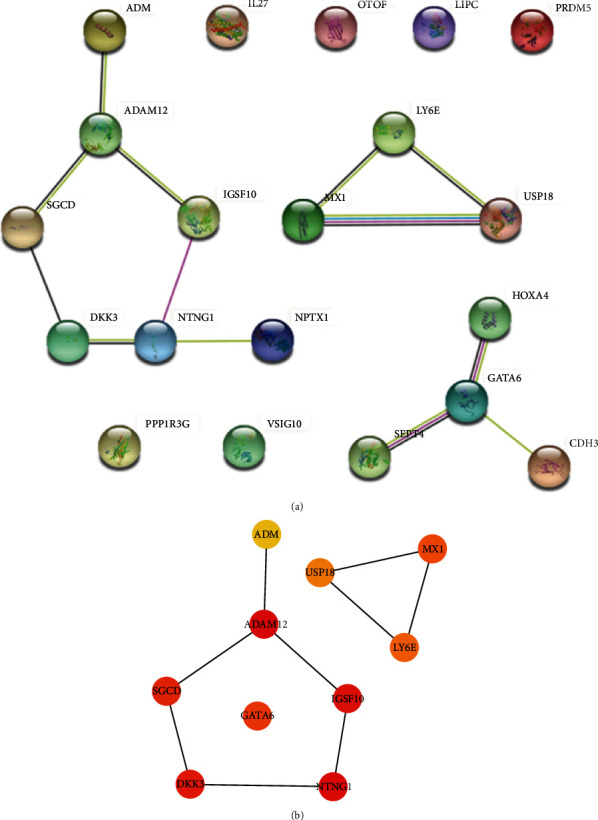
PPI networks of 20 abnormal methylation-modified DEGs in CAE. (a) PPI network of 20 abnormal methylation-modified DEGs. (b) PPI network of 10 core abnormal methylation-modified DEGs. The line between nodes means the protein interaction relationship (combined_score > 0.15).

A PPI network of 20 abnormal methylation-modified DEGs was constructed, which consists of 13 interacting gene pairs (Figure 4(a)). A total of 10 core genes were identified after the intersection of the first 10 genes of each algorithm (Figure 4(b); Table 4), including six down-regulated DEGs due to hypermethylation of NTNG1, ADAM metallopeptidase domain 12 (ADAM12), immunoglobulin superfamily member 10 (IGSF10), sarcoglycan dela (SGCD), Dickkopf WNT signaling pathway inhibitor 3 (DKK3), GATA binding protein 6 (GATA6), and four up-regulated DEGs due to hypomethylation of adrenomedullin (ADM), ubiquitin specific peptidase 18 (USP18), lymphocyte antigen 6 family member E (LY6E), and MX dynamin-like GTPase 1 (MX1). Notably, NTNG1, ADAM12, IGSF10, SGCD, DKK3, and ADM interacted with each other. A protein interaction relationship (combined_score > 0.15) was observed between USP18, LY6E, and MX1.

### 3.4. Expression Validation of Abnormal Methylation-Modified DEGs in CAE

**Figure 5:**
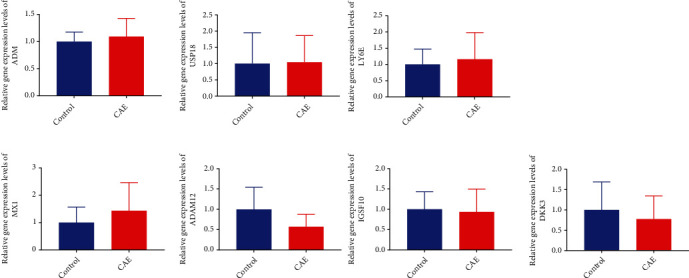
Expression validation of abnormal methylation-modified DEGs in CAE.

Seven abnormal methylation-modified DEGs (ADM, USP18, LY6E, MX1, ADAM12, IGSF10, and DKK3), were used for expression validation by RT-PCR (Figure 5). ADM, USP18, LY6E, and MX1 were up-regulated, whereas ADAM12, IGSF10, and DKK3 were down-regulated in blood samples of patients with CAE. This expression trend was consistent with the results of the informatics analysis. However, no significant difference was observed, which may be caused by the small sample size.

## 4. Discussion

GATA6 is an atherosclerosis-related transcription factor that protects against intimal hyperplasia [18, 19]. Epigenetic modifications of GATA6 are found in endothelial cells of patients with abdominal aortic aneurysms [20]. GATA6 haploin sufficiency can interrupt the extracellular matrix composition and aortic valve remodeling [21]. GATA6 affects myocardial cell proliferation and differentiation [22, 23]. Mutations in GATA6 are related to congenital heart defects, including persistent truncus arteriosus and atrial septal defects [24–26]. Here, we found that GATA6 was down-regulated due to hypermethylation in CAE. It is indicated that GATA6 may be involved in the process of atherosclerosis in patients with CAE.

USP18 is also associated with vascular injury [27, 28]. In cardiac pathophysiology, USP18 is involved in nuclear factor kappa B (NF-*κ*B) and TGF-beta-activated kinase 1 signaling pathways, which suggests that USP18 plays an important in inflammation [29]. LY6E, a plaque-related gene, is associated with inflammatory disease [30, 31]. The expression of LY6E has been found in atherosclerosis-susceptible white carneau aortic smooth muscle cells [32]. In atherosclerosis-associated cells, MX1 is involved in the type-I interferon signaling pathway [33]. It is reported that systemic lupus erythematosus patients with elevated MX1 levels exhibit declines in vascular reactivity [34]. In this study, we found that USP18, LY6E, and MX1 were up-regulated due to hypomethylation and in CAE. Moreover, there was an interaction between USP18, LY6E, and MX1 in the PPI network. It is suggested that USP18, LY6E, and MX1 may play synergistic roles in inflammation and vascular reactivity in the development of CAE.

NTNG1 is associated with vasculature in the embryo [35]. A novel locus of NTNG1 has been found in patients with cerebral atherosclerosis [36]. ADAM12 is involved in modulating endothelial cell proliferation and angiogenesis in ischemia. In mice, ADAM12 has been identified as an important gene associated with modifications of peripheral artery disease severity [37]. ADAM12 is also implicated in processes of excessive growth, such as cardiac hypertrophy [38]. IGSF10, a pro-inflammatory cytokine, is down-regulated in calcific aortic valve disease [39, 40]. SGCD is associated with aortic pathology [41]. Carriers of the Arg71Thr mutation in the SGCD gene have a late onset of dilated cardiomyopathy [42]. DKK3, a WNT signaling pathway inhibitor, regulates the migration of vascular stem cells in the vascular adventitia and affects vascular remodeling [43]. Yu et al. found that plasma levels of DKK3 were inversely related to atherosclerosis development [44]. In cardiac hypertrophy, DKK3 attenuates cardiac fibrosis by promoting angiotensinogen II degradation [45]. In mice, knockout of Dkk3 leads to greater infarcts and aggravated left ventricular function [46]. ADM, a vasoactive peptide, is involved in some cardiovascular pathophysiological processes, including vasodilatation and modulation of vascular calcification [47]. In patients with cardiovascular diseases, ADM is significantly up-regulated in the epicardial adipose tissue [48]. In the present study, we found that abnormal methylation-modified DEGs of NTNG1, ADAM12, IGSF10, SGCD, DKK3, and ADM in CAE. Furthermore, NTNG1, ADAM12, IGSF10, SGCD, DKK3, and ADM interacted with each other in the PPI network. It is indicated that these abnormal methylation-modified DEGs may be involved in inflammatory response and vascular remodeling in the process of CAE.

Based on the functional analysis of 152 DEGs, we found that cell adhesion molecules were the most significantly enriched signaling pathway. One of the DEGs, NTNG1 is involved in the signaling pathway. In addition, two significantly down-regulated signaling pathways were identified in CAE by GSEA, including O-glycan biosynthesis and the renin angiotensin system. It is reported that the levels of plasma soluble vascular cell adhesion molecule-1 levels are significantly positively associated with the total length of ectasia segments in patients with CAE [49]. Moreover, the increase of adhesion molecules facilitates the migration of neutrophils and monocytes to the vascular endothelium and promotes inflammation [49, 50]. Some genes that overexpressed in perivascular adipose tissue are involved in O- and N-glycan biosynthesis [51]. The stimulation of the renin angiotensin system leads to an enhanced inflammatory response in the vessel wall [52]. The renin angiotensin system is associated with CAE [53]. Thus it can be seen that signaling pathways of the cell adhesion molecule, O-glycan biosynthesis, and renin–angiotensin system may play important roles in the mechanism of pathology of CAE.

By integrating RNA sequencing and DNA methylation data for CAE, 9 down-regulated DEGs due to hypermethylation and 11 up-regulated DEGs due to hypomethylation were identified. In total, 10 core abnormal methylation-modified genes were further identified, including six down-regulated DEGs due to hypermethylation of NTNG1, ADAM12, IGSF10, SGCD, DKK3, GATA6, and four up-regulated DEGs due to hypomethylation of ADM, USP18, LY6E, and MX1. In addition, several signaling pathways have been identified in CAE, including cell adhesion molecule, O-glycan biosynthesis, and the renin–angiotensin system. The study may be helpful in understanding the molecular changes of genes in CAE. However, there are limitations to this study. First, the sample size for the RNA sequencing, online dataset, and RT-PCR analyses is small. A larger number of clinical samples is further required. Second, the protein levels or epigenetic landscapes of abnormal methylation-modified DEGs require further investigation. Third, the molecular mechanisms underlying CAE have not yet been investigated. Cellular experiments and animal models are required for further study. Finally, the interaction between proteins encoded by all abnormal methylation-modified DEGs is needed for further study.

## Figures and Tables

**Table 1:** Clinical information of all individuals in the RNA sequencing.

Group	Sex	Age (years old)	BMI	Creatinine (*μ*mol/L)	eGFR (mL/minutes)	Systolic pressure (mmHg)	Diastolic pressure (mmHg)	HbA1c (%)	FBG (mmol/L)	LDL-C (mmol/L)	TC (mmol/L)	Smoking history	Drinking history	Family history	Dyslipidemia	Hypertension	Diabetes
CAE	Male	66	24.7	65	96.4	145	89	5.8	7.2	2.97	3.67	Yes	Yes	No	Yes	Yes	Yes
CAE	Female	55	20.4	55.4	101.2	122	82	5.5	5.29	1.73	2.96	No	No	No	Yes	No	Yes
CAE	Female	53	26.4	73.4	80.5	101	72	5.7	5.12	2.05	3.23	No	No	No	Yes	Yes	Yes
CAE	Male	69	26.8	103	63.6	146	87	7.5	7.56	1.04	3.52	Yes	No	No	Yes	Yes	Yes
CAE	Female	72	25.8	53	91.2	166	92	5.8	4.83	2.86	4.77	No	No	No	Yes	Yes	No
Control	Female	54	19.9	64	94.3	118	77	5.8	5.8	1.8	3.01	No	No	No	No	No	No
Control	Male	45	24.2	80	96.37	141	87	5.5	6.4	2.4	3.8	Yes	No	No	Yes	No	Yes
Control	Female	59	30.1	66	84.51	124	77	5.4	5.9	3.72	5.21	No	No	No	No	Yes	No
Control	Female	65	29.7	67	82.6	140	82	5.2	5.15	2.66	4.16	No	No	No	No	No	No
Control	Male	57	24.8	60	106.1	145	97	5.9	5.97	2.62	4.25	Yes	No	No	No	Yes	No

BMI: body mass index; eGFR: epidermal growth factor receptor; HbA1c: glycosylated hemoglobin A1c; FBG: fasting blood glucose; LDL-C: low-density lipoprotein cholesterol; TC: total cholesterol. Smoking history, drinking history, and family history were based on individual statements. Dyslipidemia was diagnosed according to “2019 ESC/EAS guidelines for the management of dyslipidaemias: lipid modification to reduce cardiovascular risk, DOI: 10.1093/eurheartj/ehz455”. Hypertension was diagnosed according to “2017 ACC/AHA/AAPA/ABC/ACPM/AGS/APhA/ASH/ASPC/NMA/PCNA guideline for the prevention, detection, evaluation, and management of high blood pressure in adults, DOI: 10.1016/j.jacc.2017.11.006”. Diabetes was diagnosed according to “2. Classification and diagnosis of diabetes: standards of medical care in diabetes—2022, DOI: 10.2337/dc22-S002”.

**Table 2:** Clinical information of individuals in the RT-PCR.

Group	Gender	Age (years old)	Height (cm)	Weight (kg)	BMI	Creatinine (*μ*mol/L)	eGFR (ml/minutes)	Systolic blood pressure (mmHg)	Diastolic blood pressure (mmHg)	HbA1c (%)	Fasting blood glucose (mmol/L)	LDL-C (mmol/L)	TC (mmol/L)	Smoking history	Drinking history	Family history	Dyslipidemia	Hypertension	Diabetes
CAE	Male	48	173	75	25.1	70	106.1	127	91	5.9	4.07	1.69	3.23	Yes	Yes	No	Yes	No	No
	Male	71	180	92	28.4	80	85.1	140	93	5.6	5.47	2.17	3.56	No	Yes	No	Yes	Yes	No
	Male	45	170	90	31.1	64	112.4	133	97	5.4	5.25	4.06	6.27	No	Yes	No	Yes	No	No
	Female	63	160	66	25.8	63	90.2	128	86	5.8	5.46	1.66	3.1	No	Yes	No	No	Yes	No
	Male	46	174	84	27.7	81.6	98.9	117	73	6.6	6.3	2.34	4.08	No	Yes	No	No	Yes	No
Healthy controls	Female	39	160	63	24.6	68	97.3	117	77	5.8	7.2	2.96	4.85	No	No	No	No	Yes	No
	Male	59	165	63	23.1	85	86	120	85	5.9	4.67	3.94	5.42	No	No	No	Yes	No	No
	Female	56	174	67	22.1	49	104.6	126	77	6.9	5.51	1.6	2.89	No	No	No	No	No	Yes
	Female	60	155	55	22.8	62	93.9	109	70	7.1	5.39	2.86	4.9	No	No	No	No	No	Yes
	Female	63	160	75	29.2	65	86.8	164	97	9	8.65	2.25	3.46	No	No	No	No	Yes	Yes

CAE: coronary artery ectasia; BMI: body mass index; eGFR: epidermal growth factor receptor; HbA1c: glycosylated hemoglobin A1c; LDL-C: low-density lipoprotein cholesterol; TC: total cholesterol. Smoking history, drinking history, and family history were based on individual statements; dyslipidemia was diagnosed according to “2019 ESC/EAS Guidelines for the management of dyslipidaemias: lipid modification to reduce cardiovascular risk, DOI: 10.1093/eurheartj/ehz455”; Hypertension was diagnosed according to “2017 ACC/AHA/AAPA/ABC/ACPM/AGS/APhA/ASH/ASPC/NMA/PCNA guideline for the prevention, detection, evaluation, and management of high blood pressure in adults, DOI: 10.1016/j.jacc.2017.11.006”; Diabetes was diagnosed according to “2. Classification and diagnosis of diabetes: standards of medical care in diabetes—2022, DOI: 10.2337/dc22-S002”.

**Table 3:** KEGG analysis of 152 DEGs in RNA sequencing in CAE.

Category	Term	Count	*p*-Value	Genes
KEGG_PATHWAY	hsa04514:Cell adhesion molecules	7	0.001497	NTNG1, IGSF11, CD274, VCAM1, CDH3, SIGLEC1, and PDCD1LG2
KEGG_PATHWAY	hsa05171:Coronavirus disease-COVID-19	8	0.002355	LOC102723407, OAS1, OAS2, OAS3, MX1, ISG15, RPL3L, and C1QC
KEGG_PATHWAY	hsa05160:Hepatitis C	6	0.008094	RSAD2, OAS1, OAS2, OAS3, MX1, and IFIT1
KEGG_PATHWAY	hsa05164:Influenza A	5	0.047044	RSAD2, OAS1, OAS2, OAS3, andMX1

**Table 4:** Identification of 10 core abnormal methylation-modified genes in CAE by three algorithms.

Gene	MCC	Degree	EPC
ADAM12	3	3	2.772
NTNG1	3	3	2.733
IGSF10	2	2	2.622
SGCD	2	2	2.512
DKK3	2	2	2.482
GATA6	3	3	2.159
ADM	1	1	1.998
USP18	2	2	1.997
LY6E	2	2	1.99
MX1	2	2	1.981

MCC: maximal clique centrality; EPC: edge percolated component.

## Data Availability

All data have been deposited in the GSE222041 dataset (https://www.ncbi.nlm.nih.gov/geo/query/acc.cgi?acc=GSE222041).

## Supplementary Materials

Supplementary Materials Supplementary Table 1 DEGs in CAE.

Supplementary Materials Supplementary Table 2 Differentially methylated sites.
