# Supplementary material for: Identification of Potential Abnormal Methylation-Modified Genes in Coronary Artery Ectasia
Source: Int J Genomics. 2023 Aug 26;2023:4969605. doi: 10.1155/2023/4969605 (PMC10474963; doi:10.1155/2023/4969605)
Supplement: Supplementary Materials — Supplementary Table 1 DEGs in CAE. [file 4969605.f1.docx]

**Supplementary Table 1 DEGs in CAE**

| **Gene_id** | **BaseMean** | **lfcSE** | **Stat** | **Fold change** | **log_2_Fold Change** | **p-value** | **Up/down** |
| --- | --- | --- | --- | --- | --- | --- | --- |
| ADAM12 | 36.13102 | 0.564567 | -2.5678 | 0.366099 | -1.44969 | 0.010235 | down |
| ADM | 745.3493 | 0.339831 | 3.036519 | 2.044718 | 1.031902 | 0.002393 | up |
| AGRN | 212.9499 | 0.494808 | 2.312043 | 2.209956 | 1.144018 | 0.020775 | up |
| ARHGAP42 | 19.11891 | 0.330182 | -3.08408 | 0.493696 | -1.01831 | 0.002042 | down |
| ARHGEF10 | 63.59795 | 0.906546 | -2.68595 | 0.184931 | -2.43494 | 0.007232 | down |
| BATF2 | 231.051 | 0.438113 | 2.885106 | 2.401609 | 1.264001 | 0.003913 | up |
| BEGAIN | 17.58997 | 0.779968 | 4.024965 | 8.811243 | 3.139346 | 5.70E-05 | up |
| C1QC | 31.50101 | 0.748526 | 2.473956 | 3.609557 | 1.851822 | 0.013363 | up |
| C1QTNF4 | 10.87189 | 0.489353 | 2.855444 | 2.634118 | 1.39732 | 0.004298 | up |
| CCDC144A | 12.26955 | 1.077583 | 3.181697 | 10.767 | 3.428544 | 0.001464 | up |
| CCL4L2 | 214.9568 | 0.485726 | -2.333 | 0.455903 | -1.1332 | 0.019648 | down |
| CD274 | 471.7642 | 0.396265 | 3.013143 | 2.287867 | 1.194003 | 0.002586 | up |
| CDH3 | 1.094427 | 1.376964 | -2.00872 | 0.147018 | -2.76594 | 0.044566 | down |
| CDRT1 | 3.167051 | 0.912669 | 2.504841 | 4.87733 | 2.286092 | 0.012251 | up |
| CELF4 | 4.350779 | 1.590789 | 2.508781 | 15.89986 | 3.990942 | 0.012115 | up |
| CEMIP | 2.052951 | 1.18723 | 2.174852 | 5.987896 | 2.582049 | 0.029641 | up |
| CLEC2A | 4.572104 | 1.154293 | 2.074026 | 5.256249 | 2.394034 | 0.038077 | up |
| CMPK2 | 935.3653 | 0.665669 | 2.511082 | 3.185565 | 1.671549 | 0.012036 | up |
| COL13A1 | 19.84423 | 0.512936 | -2.00115 | 0.490914 | -1.02646 | 0.045377 | down |
| COL5A1 | 9.036754 | 0.625872 | -2.60805 | 0.322572 | -1.63231 | 0.009106 | down |
| COX6B2 | 2.597831 | 1.219636 | 2.581887 | 8.870183 | 3.148964 | 0.009826 | up |
| CPA3 | 96.95229 | 0.492483 | -2.10442 | 0.487546 | -1.03639 | 0.035342 | down |
| CXCL5 | 51.43465 | 0.447634 | 2.455637 | 2.1424 | 1.099228 | 0.014064 | up |
| DENND2B | 59.1551 | 1.097565 | 2.009869 | 4.613821 | 2.205962 | 0.044445 | up |
| DKK3 | 13.5449 | 0.699014 | -2.05949 | 0.368667 | -1.43961 | 0.039448 | down |
| DNAAF1 | 1.5894 | 1.4727 | 2.427565 | 11.91803 | 3.575074 | 0.015201 | up |
| DNTT | 2.171465 | 1.130262 | -2.24008 | 0.172913 | -2.53188 | 0.025085 | down |
| DOC2B | 20.43277 | 0.454181 | -3.82397 | 0.30004 | -1.73677 | 0.000131 | down |
| DUSP2 | 411.614 | 0.301855 | -3.70063 | 0.461034 | -1.11705 | 0.000215 | down |
| ENTPD2 | 18.38048 | 0.505394 | -2.4114 | 0.429668 | -1.21871 | 0.015891 | down |
| EREG | 18.31462 | 0.614485 | -2.29721 | 0.375895 | -1.4116 | 0.021607 | down |
| EXOC3L1 | 48.84661 | 0.466567 | 2.837483 | 2.503376 | 1.323875 | 0.004547 | up |
| FAM3B | 3.249742 | 1.764801 | -2.15933 | 0.071259 | -3.81079 | 0.030824 | down |
| FAM83E | 1.053986 | 1.454315 | -2.3564 | 0.092979 | -3.42695 | 0.018453 | down |
| FAP | 3.731909 | 0.840102 | 2.028181 | 3.257758 | 1.703879 | 0.042542 | up |
| FHIT | 110.07 | 0.607014 | -3.28634 | 0.250893 | -1.99486 | 0.001015 | down |
| FOSB | 57.83577 | 0.276045 | -4.40554 | 0.430436 | -1.21613 | 1.06E-05 | down |
| FOXG1 | 2.775404 | 1.74502 | 2.520885 | 21.09744 | 4.398996 | 0.011706 | up |
| GATA6 | 9.034647 | 0.722699 | -2.1104 | 0.347435 | -1.52519 | 0.034824 | down |
| GBP6 | 21.57525 | 0.470925 | 2.612014 | 2.345773 | 1.230064 | 0.009001 | up |
| GDF10 | 4.80748 | 0.638601 | -2.04633 | 0.40422 | -1.30679 | 0.040724 | down |
| GPM6B | 5.496435 | 0.860619 | 1.968302 | 3.235433 | 1.693959 | 0.049033 | up |
| GRIA2 | 4.16917 | 1.800577 | 2.777882 | 32.03972 | 5.00179 | 0.005471 | up |
| GSTM1 | 41.41928 | 2.901405 | -2.80284 | 0.003564 | -8.13217 | 0.005066 | down |
| HERC5 | 1613.825 | 0.614074 | 2.570287 | 2.986274 | 1.578347 | 0.010161 | up |
| HOXA4 | 4.739721 | 0.765108 | -2.88046 | 0.217055 | -2.20387 | 0.003971 | down |
| HRK | 20.39269 | 0.568554 | -2.13389 | 0.431301 | -1.21323 | 0.032852 | down |
| IFI44 | 1489.689 | 0.605985 | 2.056141 | 2.371813 | 1.24599 | 0.039769 | up |
| IFI44L | 1736.177 | 0.728943 | 2.251408 | 3.119138 | 1.641147 | 0.02436 | up |
| IFI6 | 2429.209 | 0.600844 | 2.574197 | 2.921461 | 1.54669 | 0.010047 | up |
| IFIT1 | 3445.594 | 0.71354 | 2.509275 | 3.459271 | 1.790468 | 0.012098 | up |
| IFIT2 | 7015.676 | 0.437107 | 3.019404 | 2.49632 | 1.319803 | 0.002533 | up |
| IFIT3 | 6108.948 | 0.669685 | 2.416659 | 3.070342 | 1.618399 | 0.015664 | up |
| IFITM10 | 15.84839 | 0.449832 | 2.516136 | 2.191379 | 1.131839 | 0.011865 | up |
| IFITM3 | 14681.51 | 0.615413 | 2.224739 | 2.583154 | 1.369133 | 0.026099 | up |
| IGSF10 | 5.08837 | 0.844078 | -2.10155 | 0.292423 | -1.77387 | 0.035593 | down |
| IGSF11 | 1.211006 | 1.35437 | -2.14321 | 0.133721 | -2.9027 | 0.032096 | down |
| IL17C | 4.082563 | 0.668561 | -2.06932 | 0.383296 | -1.38347 | 0.038516 | down |
| IL27 | 12.87961 | 0.542749 | 2.03005 | 2.146234 | 1.101808 | 0.042351 | up |
| IL3RA-2 | 20.17265 | 0.70396 | 1.966992 | 2.611148 | 1.384684 | 0.049184 | up |
| INSYN2B | 3.396848 | 0.888473 | 2.004642 | 3.436811 | 1.781071 | 0.045001 | up |
| ISG15 | 1728.252 | 0.678479 | 2.67134 | 3.512374 | 1.812447 | 0.007555 | up |
| KIR2DL3 | 126.9886 | 0.417929 | -2.50295 | 0.484291 | -1.04605 | 0.012316 | down |
| KLHL33 | 8.55364 | 0.540555 | 2.585118 | 2.634262 | 1.397399 | 0.009735 | up |
| KLRC2 | 124.2451 | 0.446079 | -2.40266 | 0.475733 | -1.07178 | 0.016276 | down |
| KLRC4-KLRK1 | 12.7991 | 0.426216 | -2.81958 | 0.434748 | -1.20175 | 0.004809 | down |
| LAMP3 | 99.94386 | 0.542912 | 3.098563 | 3.209274 | 1.682247 | 0.001945 | up |
| LGALSL | 251.2478 | 0.288689 | 4.076004 | 2.260584 | 1.176696 | 4.58E-05 | up |
| LINGO2 | 23.62497 | 0.529378 | -2.1772 | 0.449827 | -1.15256 | 0.029466 | down |
| LIPC | 16.13324 | 0.643087 | 2.471072 | 3.008645 | 1.589114 | 0.013471 | up |
| LOC102723407 | 11.43988 | 0.790727 | 4.204468 | 10.01844 | 3.324586 | 2.62E-05 | up |
| LOC105372412 | 26.17338 | 0.399984 | 3.798765 | 2.866811 | 1.519447 | 0.000145 | up |
| LOC107987285 | 6.476601 | 0.731166 | 2.168227 | 3.000774 | 1.585335 | 0.030141 | up |
| LOXHD1 | 119.4028 | 0.457502 | 2.545692 | 2.241802 | 1.164659 | 0.010906 | up |
| LY6E | 4447.286 | 0.624438 | 1.963028 | 2.338833 | 1.225789 | 0.049643 | up |
| MAP3K7CL | 445.7496 | 0.410488 | 3.22338 | 2.502134 | 1.323159 | 0.001267 | up |
| MS4A2 | 62.72227 | 0.528601 | -2.44128 | 0.408819 | -1.29047 | 0.014635 | down |
| MTRNR2L8 | 4.364341 | 0.817802 | 2.225279 | 3.530413 | 1.819837 | 0.026062 | up |
| MX1 | 4772.18 | 0.467995 | 3.119768 | 2.75115 | 1.460035 | 0.00181 | up |
| MYBPH | 32.6193 | 0.711347 | -2.49034 | 0.292904 | -1.7715 | 0.012762 | down |
| MYOM2 | 1029.445 | 0.90785 | 3.54899 | 9.330484 | 3.221952 | 0.000387 | up |
| NEBL | 22.1191 | 1.423002 | 2.031557 | 7.417385 | 2.890911 | 0.042199 | up |
| NEFL | 53.25895 | 0.603161 | 2.012368 | 2.319447 | 1.213781 | 0.044181 | up |
| NEU4 | 2.330925 | 1.099064 | 2.289238 | 5.720009 | 2.516017 | 0.022066 | up |
| NEXMIF | 10.89794 | 0.702943 | -2.04206 | 0.369731 | -1.43545 | 0.041146 | down |
| NFILZ | 25.27986 | 0.530728 | -3.14144 | 0.314853 | -1.66725 | 0.001681 | down |
| NPIPA7 | 40.11756 | 0.343408 | -2.97267 | 0.492829 | -1.02084 | 0.002952 | down |
| NPTX1 | 37.49878 | 1.158724 | 2.568013 | 7.865938 | 2.975619 | 0.010228 | up |
| NT5M | 133.4476 | 0.472127 | 2.786128 | 2.488724 | 1.315406 | 0.005334 | up |
| NTNG1 | 1.333028 | 1.460141 | -2.12324 | 0.11661 | -3.10023 | 0.033734 | down |
| OAS1 | 3510.964 | 0.476645 | 2.50312 | 2.286434 | 1.193099 | 0.01231 | up |
| OAS2 | 3741.28 | 0.401739 | 2.703901 | 2.123231 | 1.086261 | 0.006853 | up |
| OAS3 | 6266.334 | 0.74043 | 2.416046 | 3.455542 | 1.788912 | 0.01569 | up |
| OASL | 1533.866 | 0.431933 | 3.029592 | 2.476976 | 1.30858 | 0.002449 | up |
| OLFM1 | 104.2416 | 0.376315 | 2.66952 | 2.006361 | 1.004581 | 0.007596 | up |
| OR7D2 | 7.065086 | 1.44449 | -2.8115 | 0.059905 | -4.06118 | 0.004931 | down |
| OTOF | 120.7155 | 1.064742 | 2.3072 | 5.489112 | 2.456573 | 0.021044 | up |
| PAGE2B | 30.64541 | 0.494052 | 2.59019 | 2.427867 | 1.279689 | 0.009592 | up |
| PCDHGA7 | 3.499933 | 0.854374 | -2.60867 | 0.213339 | -2.22878 | 0.009089 | down |
| PDCD1LG2 | 44.43347 | 0.443728 | 3.593733 | 3.020195 | 1.594642 | 0.000326 | up |
| PHYHIP | 7.981906 | 3.030869 | 2.164342 | 94.34252 | 6.559836 | 0.030438 | up |
| PLEKHG4B | 1.760277 | 1.461335 | 2.567079 | 13.46705 | 3.751362 | 0.010256 | up |
| PLP1 | 11.55215 | 1.393791 | 3.588776 | 32.04449 | 5.002005 | 0.000332 | up |
| PLPP2 | 5.236196 | 0.760966 | -3.41291 | 0.16527 | -2.59711 | 0.000643 | down |
| PLPPR4 | 2.380683 | 1.076908 | -2.05952 | 0.214953 | -2.21791 | 0.039445 | down |
| PM20D1 | 19.64001 | 0.339029 | -2.97041 | 0.49756 | -1.00706 | 0.002974 | down |
| PNMA6A | 8.02315 | 1.282135 | 2.700006 | 11.01786 | 3.461772 | 0.006934 | up |
| POLR2J3 | 49.03655 | 0.316737 | 3.219343 | 2.027476 | 1.019685 | 0.001285 | up |
| PPP1R3G | 5.939996 | 0.612368 | -2.19219 | 0.394357 | -1.34243 | 0.028366 | down |
| PRAME | 2.784562 | 1.693992 | -2.10029 | 0.084913 | -3.55787 | 0.035703 | down |
| PRDM5 | 70.6032 | 0.550134 | 2.184188 | 2.299942 | 1.201597 | 0.028948 | up |
| PROM2 | 8.934512 | 0.830826 | -2.45687 | 0.242956 | -2.04123 | 0.014015 | down |
| PROS1 | 45.23229 | 0.50359 | 2.006294 | 2.0144 | 1.01035 | 0.044825 | up |
| RAMP1 | 15.8139 | 0.556928 | -2.18563 | 0.430105 | -1.21724 | 0.028843 | down |
| RAMP3 | 11.16325 | 0.914648 | 1.964784 | 3.475176 | 1.797086 | 0.049439 | up |
| RAPGEF3 | 32.08494 | 0.885018 | 2.718648 | 5.30022 | 2.406052 | 0.006555 | up |
| RBPMS2 | 18.02342 | 0.584481 | 2.987609 | 3.354738 | 1.7462 | 0.002812 | up |
| RGPD1 | 11.17326 | 0.720929 | -2.75713 | 0.252142 | -1.98769 | 0.005831 | down |
| RGPD2 | 26.58492 | 0.594036 | -2.91239 | 0.301439 | -1.73006 | 0.003587 | down |
| RGPD3 | 19.3048 | 0.47737 | -2.38648 | 0.454001 | -1.13923 | 0.017011 | down |
| RNF17 | 3.521612 | 1.904487 | 2.186593 | 17.93044 | 4.164339 | 0.028772 | up |
| RPL3L | 2.919988 | 1.090462 | -1.97926 | 0.224019 | -2.1583 | 0.047787 | down |
| RSAD2 | 2848.648 | 0.853992 | 2.521867 | 4.449536 | 2.153655 | 0.011673 | up |
| RSPH9 | 20.40972 | 0.597285 | 2.336462 | 2.630858 | 1.395534 | 0.019467 | up |
| RUNDC3B | 2.676408 | 0.952668 | -2.52819 | 0.188348 | -2.40853 | 0.011465 | down |
| SCGB1C2 | 1.852433 | 1.136802 | 2.20477 | 5.681953 | 2.506387 | 0.02747 | up |
| SEPTIN3 | 5.749396 | 0.610224 | 2.181289 | 2.515901 | 1.331075 | 0.029162 | up |
| SEPTIN4 | 53.92236 | 0.470825 | 2.412556 | 2.197543 | 1.135892 | 0.015841 | up |
| SERPING1 | 1020.478 | 0.676546 | 2.269872 | 2.899235 | 1.535673 | 0.023215 | up |
| SGCD | 5.41706 | 0.706567 | -2.94499 | 0.236378 | -2.08083 | 0.00323 | down |
| SIGLEC1 | 870.7223 | 0.745656 | 2.74527 | 4.132534 | 2.047027 | 0.006046 | up |
| SLC12A8 | 5.249209 | 0.567705 | 2.297565 | 2.469705 | 1.304339 | 0.021587 | up |
| SLC22A1 | 63.9104 | 0.35472 | 2.833468 | 2.007064 | 1.005087 | 0.004605 | up |
| SLC5A11 | 6.763154 | 1.024011 | -2.39722 | 0.182405 | -2.45478 | 0.01652 | down |
| SLCO2B1 | 2.063889 | 1.141177 | 2.034707 | 5.000112 | 2.32196 | 0.04188 | up |
| SMIM11A | 12.17041 | 1.106772 | 2.110722 | 5.049317 | 2.336088 | 0.034796 | up |
| SNAP25 | 11.13925 | 1.27359 | 2.81127 | 11.96217 | 3.580407 | 0.004935 | up |
| SNX7 | 2.01625 | 1.088451 | -2.21894 | 0.187478 | -2.41521 | 0.026491 | down |
| TBC1D3 | 13.06574 | 0.903685 | 2.203188 | 3.975092 | 1.990988 | 0.027581 | up |
| TDRD9 | 74.87421 | 0.293475 | 3.902212 | 2.211769 | 1.145201 | 9.53E-05 | up |
| TFCP2L1 | 38.78715 | 0.495566 | -2.62658 | 0.405664 | -1.30164 | 0.008625 | down |
| TMEM119 | 10.93537 | 0.506164 | 2.553384 | 2.449406 | 1.292432 | 0.010668 | up |
| TREML4 | 18.58696 | 3.013673 | -1.98945 | 0.015673 | -5.99557 | 0.046651 | down |
| TRPV4 | 16.22052 | 0.481849 | 2.715138 | 2.476472 | 1.308286 | 0.006625 | up |
| TSGA10IP | 3.460425 | 0.866533 | 2.33798 | 4.072567 | 2.025938 | 0.019388 | up |
| TTLL9 | 1.618299 | 1.368411 | -1.96127 | 0.155629 | -2.68382 | 0.049848 | down |
| USP18 | 137.1008 | 0.453582 | 2.732737 | 2.361202 | 1.239521 | 0.006281 | up |
| VCAM1 | 14.29125 | 0.711395 | -2.45706 | 0.297726 | -1.74794 | 0.014008 | down |
| VSIG10 | 59.73039 | 0.256411 | 4.230292 | 2.120926 | 1.084694 | 2.33E-05 | up |
| XCL1 | 100.5258 | 0.410683 | -4.04072 | 0.316559 | -1.65946 | 5.33E-05 | down |
| ZNF534 | 1.212603 | 1.597321 | -2.28417 | 0.07974 | -3.64856 | 0.022361 | down |
| ZP1 | 2.081201 | 1.656752 | 2.032576 | 10.32073 | 3.367474 | 0.042095 | up |
