# Supplementary material for: Identification of Potential Abnormal Methylation-Modified Genes in Coronary Artery Ectasia
Source: Int J Genomics. 2023 Aug 26;2023:4969605. doi: 10.1155/2023/4969605 (PMC10474963; doi:10.1155/2023/4969605)
Supplement: Supplementary Materials — Supplementary Table 2 Differentially methylated sites. [file 4969605.f2.pdf]

gene	
MARCHF4	hyper
MARCHF8	hyper
SEPTIN4	hypo
SEPTIN8	hyper
SEPTIN9	hypo
SEPTIN10	hyper
SELENOF	hyper
A2LD1	hypo
A4GALT	hypo
AADACL3	hyper
AAK1	hyper
AARS	hyper
AASDHPPT	hypo
AASS	hyper
AATK	hypo
ABCA11P	hyper
ABCA17P	hyper
ABCA4	hyper
ABCA7	hypo
ABCB1	hyper
ABCB4	hyper
ABCB8	hypo
ABCC1	hyper
ABCC10	hypo
ABCC4	hypo
ABCC8	hyper
ABCD2	hyper
ABCF1	hypo
ABCG1	hyper
ABCG2	hypo
ABCG8	hypo
ABHD4	hyper
ABI3	hypo
ABLIM1	hyper
ABLIM3	hyper
ABO	hyper
ABTB2	hypo
ACAA1	hypo
ACAD10	hypo
ACADL	hypo
ACAP1	hyper
ACAT2	hypo
ACBD3	hypo
ACCS	hypo
ACHE	hypo
ACLY	hyper
ACO1	hypo
ACOT8	hypo
ACOXL	hypo
ACP1	hyper
ACP6	hypo
ACPL2	hyper
ACPT	hypo
ACRV1	hypo
ACSF3	hypo
ACSL3	hyper
ACTA2	hyper

ACTC1	hyper
ACTL7B	hypo
ACTN3	hypo
ACVRL1	hyper
ADAM12	hyper
ADAM15	hypo
ADAM22	hyper
ADAM28	hyper
ADAM30	hyper
ADAM32	hyper
ADAM7	hyper
ADAMTS1	hyper
ADAMTS10	hyper
ADAMTS13	hyper
ADAMTS16	hyper
ADAMTS18	hyper
ADAMTS6	hypo
ADAMTSL2	hypo
ADC	hypo
ADCK1	hyper
ADCK5	hyper
ADCY1	hyper
ADCY10	hypo
ADCY8	hyper
ADCY9	hyper
ADCYAP1	hypo
ADD2	hyper
ADI1	hyper
ADIPOR2	hyper
ADM	hypo
ADM2	hyper
ADNP	hypo
ADO	hypo
ADORA1	hypo
ADPRH	hypo
ADPRHL1	hypo
ADSL	hypo
AEN	hypo
AFAP1L1	hypo
AFF1	hyper
AFF3	hypo
AFG3L2	hypo
AFTPH	hyper
AGA	hypo
AGAP2	hyper
AGAP3	hyper
AGBL1	hypo
AGBL3	hyper
AGPAT3	hyper
AGPAT4	hyper
AGXT2L1	hypo
AHCTF1	hypo
AHCY	hyper
AHSA2	hypo
AIDA	hypo
AIFM2	hypo
AIMP1	hypo
AIRE	hypo

AKAP11	hypo
AKAP13	hyper
AKAP9	hypo
AKD1	hyper
AKNAD1	hyper
ALAD	hyper
ALDH1A2	hypo
ALDH1A3	hyper
ALDH1B1	hyper
ALDH2	hypo
ALDH3A1	hypo
ALDH3A2	hyper
ALDH3B2	hypo
ALDH5A1	hyper
ALDOA	hyper
ALG10B	hypo
ALG14	hypo
ALG3	hypo
ALKBH6	hyper
ALKBH7	hyper
ALOX15	hyper
ALOXE3	hypo
ALPK1	hyper
ALPL	hyper
ALPP	hypo
ALPPL2	hypo
ALS2	hypo
ALS2CL	hypo
ALX3	hypo
ALX4	hyper
AMDHD1	hyper
AMDHD2	hypo
AMH	hyper
AMN	hyper
ANG	hyper
ANGEL2	hypo
ANGPTL6	hyper
ANK1	hypo
ANKAR	hypo
ANKK1	hyper
ANKLE1	hyper
ANKRD17	hyper
ANKRD24	hypo
ANKRD26P1	hyper
ANKRD33B	hyper
ANKRD34A	hyper
ANKRD43	hyper
ANKRD44	hypo
ANKRD53	hypo
ANKRD54	hypo
ANKRD9	hyper
ANKS1A	hyper
ANKS3	hypo
ANKS6	hypo
ANLN	hypo
ANO10	hypo
ANO5	hyper
ANO7	hyper

ANO9	hypo
ANP32A	hyper
ANP32E	hypo
ANTXR2	hypo
ANUBL1	hyper
ANXA13	hyper
ANXA4	hyper
AOC3	hypo
AP1AR	hypo
AP1S3	hypo
AP2B1	hypo
AP3B2	hypo
APC2	hyper
APCDD1	hyper
APEH	hypo
APLP2	hypo
APOB48R	hyper
APOC4	hypo
AQP11	hyper
AQP2	hypo
AQP4	hyper
ARC	hyper
ARCN1	hyper
ARF5	hyper
ARF6	hyper
ARFGAP3	hyper
ARFIP1	hyper
ARHGAP10	hypo
ARHGAP19	hyper
ARHGAP22	hyper
ARHGAP27	hyper
ARHGAP9	hyper
ARHGDIG	hyper
ARHGEF15	hypo
ARHGEF16	hypo
ARHGEF17	hyper
ARHGEF18	hyper
ARID1A	hyper
ARID3A	hyper
ARID3B	hyper
ARID5B	hypo
ARL2BP	hypo
ARL4A	hyper
ARL4C	hypo
ARL5B	hypo
ARL6IP1	hypo
ARL6IP5	hypo
ARL8A	hypo
ARMC1	hyper
ARNTL2	hypo
ARPC1B	hyper
ARPC4	hypo
ARPC5L	hypo
ARRDC4	hyper
ART3	hyper
ART5	hypo
AS3MT	hyper
ASAM	hypo

ASAP1	hyper
ASAP1IT1	hyper
ASB5	hyper
ASB6	hyper
ASCL2	hyper
ASPHD2	hypo
ASPSCR1	hypo
ATCAY	hyper
ATG12	hyper
ATG2A	hypo
ATG4B	hyper
ATG9B	hypo
ATL1	hyper
ATM	hypo
ATMIN	hyper
ATOH8	hyper
ATP10D	hypo
ATP13A2	hypo
ATP13A3	hypo
ATP1B2	hyper
ATP2A3	hyper
ATP2C2	hyper
ATP4B	hypo
ATP5B	hypo
ATP5D	hyper
ATP6AP1L	hypo
ATP6V0C	hyper
ATP6V0E2	hyper
ATP6V1C1	hypo
ATP8A2	hyper
ATP8B1	hyper
ATP8B3	hyper
ATXN1	hypo
ATXN3	hypo
ATXN7L1	hyper
AURKAIP1	hyper
AVL9	hyper
AVP	hyper
AVPR1A	hyper
AXIN2	hypo
B3GALT4	hyper
B3GNT2	hypo
B3GNT8	hyper
B4GALNT1	hypo
B4GALNT2	hyper
B4GALNT4	hyper
B4GALT2	hyper
B4GALT5	hyper
BACH1	hyper
BACH2	hyper
BAD	hypo
BAHCC1	hyper
BAHD1	hypo
BAI2	hyper
BAI3	hyper
BAIAP2	hyper
BAIAP2L2	hypo
BANF1	hypo

BARHL1	hyper
BARHL2	hyper
BARX2	hypo
BAT1	hypo
BAT4	hyper
BAZ2A	hyper
BAZ2B	hyper
BBS5	hyper
BCAP29	hyper
BCAR1	hypo
BCAS3	hyper
BCAT1	hyper
BCDIN3D	hyper
BCKDHA	hypo
BCL10	hyper
BCL6	hypo
BCL9L	hyper
BCO2	hyper
BDH1	hyper
BDKRB2	hyper
BEND3	hypo
BEST1	hypo
BFAR	hypo
BFSP1	hyper
BHLHE41	hyper
BHMT2	hyper
BICC1	hyper
BICD1	hyper
BIK	hypo
BIRC2	hypo
BLNK	hypo
BLOC1S1	hypo
BLOC1S2	hypo
BLOC1S3	hyper
BMF	hypo
BMI1	hyper
BMP1	hyper
BMP10	hypo
BMP3	hyper
BMP7	hyper
BMP8A	hyper
BMP8B	hyper
BMPER	hyper
BMPR1A	hypo
BMPR2	hypo
BMS1P4	hypo
BNC2	hyper
BNIP3	hyper
BOP1	hypo
BPI	hypo
BPIL2	hypo
BPNT1	hypo
BPTF	hyper
BRAF	hyper
BRCA1	hyper
BRD2	hyper
BRD9	hypo
BRF1	hypo

BRF2	hyper
BRIX1	hypo
BRMS1	hyper
BRMS1L	hypo
BRUNOL4	hyper
BRUNOL6	hypo
BSG	hyper
BSN	hypo
BTBD12	hyper
BTD	hyper
BTF3L4	hyper
BTNL8	hypo
BTNL9	hypo
BUB1	hypo
BUD31	hyper
BZRAP1	hyper
C10orf10	hyper
C10orf107	hyper
C10orf11	hypo
C10orf110	hyper
C10orf116	hyper
C10orf140	hyper
C10orf2	hyper
C10orf25	hypo
C10orf27	hypo
C10orf46	hypo
C10orf54	hyper
C10orf62	hyper
C10orf67	hyper
C11orf10	hyper
C11orf30	hyper
C11orf35	hyper
C11orf60	hypo
C11orf65	hypo
C11orf68	hypo
C11orf75	hypo
C11orf86	hypo
C11orf93	hyper
C12orf34	hypo
C12orf49	hypo
C12orf61	hypo
C12orf62	hypo
C13orf15	hypo
C13orf16	hypo
C13orf18	hypo
C13orf28	hyper
C13orf35	hypo
C13orf36	hyper
C14orf105	hyper
C14orf115	hypo
C14orf135	hypo
C14orf182	hyper
C14orf184	hypo
C14orf23	hyper
C14orf37	hypo
C14orf38	hyper
C14orf68	hypo
C14orf93	hyper

C15orf27	hyper
C15orf33	hypo
C15orf60	hyper
C16orf38	hyper
C16orf45	hyper
C16orf53	hyper
C16orf55	hypo
C16orf58	hypo
C16orf62	hyper
C16orf63	hypo
C16orf7	hyper
C16orf70	hyper
C17orf104	hyper
C17orf39	hypo
C17orf49	hypo
C17orf57	hypo
C17orf59	hypo
C17orf70	hypo
C17orf79	hyper
C17orf97	hyper
C18orf1	hyper
C18orf34	hypo
C18orf54	hypo
C19orf21	hypo
C19orf26	hyper
C19orf29	hypo
C19orf30	hyper
C19orf35	hypo
C19orf44	hypo
C19orf50	hypo
C19orf51	hyper
C19orf61	hypo
C1D	hypo
C1orf105	hypo
C1orf109	hyper
C1orf114	hyper
C1orf130	hyper
C1orf151	hyper
C1orf158	hypo
C1orf172	hyper
C1orf175	hyper
C1orf192	hyper
C1orf213	hyper
C1orf228	hypo
C1orf229	hyper
C1orf43	hypo
C1orf57	hyper
C1orf86	hyper
C1orf88	hyper
C1orf92	hypo
C1orf94	hypo
C1QB	hypo
C1QL1	hyper
C1QL3	hypo
C1QTNF5	hypo
C1QTNF8	hyper
C1QTNF9	hyper
C1RL	hypo

C1S	hypo
C2	hyper
C20orf118	hypo
C20orf152	hypo
C20orf177	hypo
C20orf194	hyper
C20orf24	hypo
C20orf29	hypo
C20orf4	hypo
C20orf43	hyper
C20orf94	hypo
C21orf45	hyper
C21orf57	hypo
C21orf66	hypo
C21orf82	hyper
C22orf23	hypo
C22orf24	hypo
C22orf40	hypo
C2CD2	hyper
C2CD4C	hypo
C2orf3	hypo
C2orf39	hyper
C2orf40	hyper
C2orf42	hyper
C2orf49	hyper
C2orf55	hyper
C2orf60	hypo
C2orf61	hypo
C2orf63	hypo
C2orf83	hypo
C2orf86	hyper
C2orf89	hyper
C3	hypo
C3orf14	hyper
C3orf17	hyper
C3orf21	hypo
C3orf31	hypo
C3orf39	hyper
C3orf45	hypo
C3orf54	hypo
C3orf57	hyper
C3orf58	hypo
C3orf67	hyper
C3orf70	hyper
C3orf75	hypo
C4orf19	hyper
C4orf23	hyper
C4orf31	hypo
C4orf35	hyper
C4orf39	hypo
C4orf41	hypo
C4orf42	hyper
C4orf43	hypo
C4orf44	hypo
C4orf49	hyper
C5orf22	hypo
C5orf49	hyper
C5orf52	hypo

C6orf10	hypo
C6orf130	hyper
C6orf132	hypo
C6orf136	hyper
C6orf141	hypo
C6orf142	hyper
C6orf146	hyper
C6orf147	hyper
C6orf153	hypo
C6orf162	hypo
C6orf221	hyper
C6orf25	hypo
C6orf26	hyper
C6orf35	hyper
C6orf48	hypo
C6orf62	hypo
C6orf72	hyper
C6orf89	hypo
C7orf16	hypo
C7orf20	hypo
C7orf23	hyper
C7orf26	hyper
C7orf27	hypo
C7orf31	hypo
C7orf47	hypo
C7orf50	hyper
C7orf52	hyper
C7orf57	hyper
C7orf64	hyper
C7orf72	hypo
C8orf4	hyper
C8orf40	hyper
C8orf45	hypo
C8orf59	hypo
C8orf73	hyper
C8orf75	hyper
C8ORFK29	hypo
C9orf116	hyper
C9orf122	hyper
C9orf23	hypo
C9orf3	hypo
C9orf43	hypo
C9orf47	hyper
C9orf68	hyper
C9orf89	hypo
C9orf98	hypo
CA12	hypo
CA14	hyper
CA3	hyper
CA5A	hyper
CAB39	hypo
CABLES1	hyper
CABLES2	hypo
CACNA1A	hypo
CACNA1C	hyper
CACNA1D	hypo
CACNA1E	hyper
CACNA1G	hypo

CACNA1I	hyper
CACNA2D3	hypo
CACNB4	hyper
CACNG2	hyper
CACNG3	hyper
CACNG8	hyper
CACYBP	hypo
CADM1	hyper
CADM2	hyper
CALCB	hyper
CALCOCO1	hyper
CALCOCO2	hyper
CALHM1	hyper
CALHM2	hyper
CALM1	hypo
CALR	hypo
CAMK2A	hypo
CAMK2B	hyper
CAMKV	hyper
CAMLG	hyper
CAND2	hypo
CANT1	hyper
CAP2	hyper
CAPN1	hypo
CAPN10	hypo
CAPN9	hypo
CAPS	hypo
CAPS2	hypo
CARD11	hyper
CARD14	hyper
CARKD	hyper
CARTPT	hyper
CASC2	hypo
CASKIN1	hypo
CASP7	hypo
CASQ2	hypo
CASZ1	hypo
CAT	hypo
CATSPER1	hypo
CATSPERG	hypo
CAV1	hypo
CBFA2T3	hyper
CBLL1	hypo
CBS	hyper
CBX4	hyper
CCAR1	hypo
CCDC102B	hypo
CCDC103	hypo
CCDC105	hypo
CCDC108	hypo
CCDC109B	hypo
CCDC110	hypo
CCDC112	hyper
CCDC116	hypo
CCDC117	hypo
CCDC126	hypo
CCDC130	hyper
CCDC135	hyper

CCDC141	hypo
CCDC146	hyper
CCDC147	hyper
CCDC15	hypo
CCDC153	hypo
CCDC162	hypo
CCDC163P	hypo
CCDC17	hyper
CCDC18	hypo
CCDC3	hyper
CCDC37	hypo
CCDC46	hypo
CCDC48	hyper
CCDC53	hyper
CCDC57	hypo
CCDC70	hypo
CCDC76	hyper
CCDC85C	hypo
CCDC88C	hypo
CCDC9	hyper
CCHCR1	hypo
CCKBR	hyper
CCL19	hyper
CCL28	hyper
CCNA2	hypo
CCNB1	hypo
CCND1	hyper
CCND2	hyper
CCND3	hypo
CCNF	hypo
CCNG1	hyper
CCNJ	hyper
CCR10	hyper
CD160	hyper
CD163L1	hypo
CD180	hyper
CD19	hypo
CD200R1	hyper
CD22	hypo
CD248	hypo
CD274	hyper
CD276	hypo
CD33	hyper
CD36	hypo
CD38	hypo
CD40	hyper
CD52	hypo
CD59	hypo
CD8A	hyper
CD9	hyper
CD97	hypo
CDC25A	hyper
CDC25C	hypo
CDC40	hypo
CDC42	hypo
CDC42BPA	hyper
CDC42EP3	hyper
CDC42EP5	hyper

CDC6	hypo
CDCA7L	hyper
CDCA8	hyper
CDGAP	hyper
CDH1	hyper
CDH11	hyper
CDH12	hyper
CDH18	hyper
CDH2	hyper
CDH23	hypo
CDH3	hyper
CDH4	hypo
CDH6	hyper
CDK13	hyper
CDK15	hyper
CDK17	hyper
CDK18	hyper
CDK2AP2	hypo
CDK5R1	hyper
CDK5R2	hyper
CDK5RAP1	hyper
CDK5RAP2	hypo
CDKL2	hyper
CDKN1B	hypo
CDKN1C	hyper
CDKN3	hyper
CDR2L	hyper
CEACAM5	hypo
CEACAM8	hyper
CEBPZ	hypo
CELSR2	hypo
CELSR3	hyper
CENPF	hyper
CENPQ	hypo
CENPT	hypo
CENPV	hyper
CEP120	hypo
CEP164	hypo
CEP250	hypo
CEP55	hyper
CEP57	hypo
CEP78	hyper
CERKL	hyper
CES4	hyper
CGREF1	hyper
CHCHD3	hyper
CHD1L	hyper
CHD5	hypo
CHFR	hyper
CHGB	hypo
CHIA	hyper
CHID1	hyper
CHL1	hyper
CHMP4C	hypo
CHMP6	hyper
CHN1	hypo
CHP	hypo
CHRDL2	hyper

CHRM1	hypo
CHRM2	hyper
CHRNA2	hypo
CHRNE	hypo
CHST11	hypo
CHST13	hyper
CHSY1	hypo
CHTF18	hyper
CHTF8	hypo
CIAO1	hypo
CIB4	hypo
CIITA	hypo
CILP	hypo
CILP2	hyper
CITED2	hypo
CITED4	hyper
CKAP2	hypo
CKB	hyper
CLASP1	hypo
CLC	hypo
CLCA1	hyper
CLCA2	hyper
CLCA4	hyper
CLCN1	hyper
CLCN7	hyper
CLCNKA	hypo
CLDN1	hypo
CLDN11	hyper
CLDN12	hypo
CLDN16	hypo
CLDN6	hyper
CLDN7	hypo
CLDND1	hyper
CLEC16A	hyper
CLEC2L	hyper
CLEC3B	hyper
CLEC4GP1	hypo
CLEC9A	hyper
CLIC1	hyper
CLIC5	hyper
CLOCK	hyper
CLPTM1L	hyper
CLRN2	hypo
CLSTN2	hyper
CLVS1	hyper
CLVS2	hyper
CLYBL	hyper
CMIP	hyper
CMPK1	hypo
CMTM1	hyper
CMTM2	hyper
CNDP1	hypo
CNDP2	hyper
CNGB1	hypo
CNN1	hypo
CNNM2	hyper
CNOT10	hypo
CNOT2	hypo

CNOT6L	hypo
CNP	hyper
CNTD2	hyper
CNTN5	hyper
CNTN6	hypo
CNTNAP4	hypo
CNTNAP5	hyper
COASY	hypo
COBL	hyper
COBRA1	hypo
COL11A1	hyper
COL18A1	hyper
COL19A1	hyper
COL1A2	hyper
COL24A1	hyper
COL25A1	hyper
COL2A1	hyper
COL4A2	hyper
COL4A3	hyper
COL5A2	hypo
COL5A3	hyper
COL6A1	hyper
COL7A1	hypo
COL9A2	hypo
COL9A3	hyper
COLEC10	hyper
COLEC11	hypo
COMMD3	hyper
COMMD8	hypo
COMP	hyper
COMTD1	hypo
COPB1	hypo
COPS3	hyper
COPS4	hyper
COPS6	hypo
COPZ1	hypo
COQ10B	hypo
COQ5	hypo
CORIN	hyper
CORO1B	hypo
CORO1C	hyper
CORO2B	hypo
CORO6	hypo
COTL1	hyper
COX16	hypo
COX19	hypo
COX4NB	hyper
COX5A	hypo
COX6A1	hypo
COX7B2	hyper
CP110	hyper
CPA2	hyper
CPEB1	hypo
CPEB3	hypo
CPLX1	hyper
CPLX4	hypo
CPNE6	hyper
CPNE7	hyper

CPNE8	hyper
CPNE9	hyper
CPOX	hypo
CPSF1	hypo
CPSF3	hyper
CPSF4	hyper
CPSF4L	hypo
CPVL	hyper
CPZ	hyper
CR2	hypo
CRABP1	hyper
CRADD	hypo
CRB1	hyper
CRB2	hypo
CREB1	hypo
CREB3L3	hypo
CREB5	hypo
CREBZF	hypo
CRELD2	hyper
CREM	hypo
CRH	hyper
CRHR1	hypo
CRIPT	hypo
CRISPLD2	hyper
CRLF1	hyper
CRLF3	hypo
CRLS1	hypo
CRTAC1	hyper
CRTC3	hypo
CRY1	hypo
CRY2	hyper
CRYBB2	hyper
CRYBB3	hyper
CRYGB	hypo
CRYL1	hypo
CS	hypo
CSAD	hypo
CSF3R	hypo
CSGALNACT1	hyper
CSGALNACT2	hypo
CSMD1	hypo
CSNK1D	hyper
CSNK1G1	hypo
CSNK1G3	hypo
CSNK2A1	hyper
CSNK2A2	hyper
CST8	hypo
CST9L	hyper
CSTF1	hyper
CTCFL	hyper
CTDP1	hyper
CTDSPL	hyper
CTH	hypo
CTNNA1	hypo
CTNNA2	hyper
CTNNBL1	hyper
CTNND2	hypo
CTPS	hypo

CTR9	hypo
CTRB1	hypo
CTSF	hypo
CTSG	hyper
CTU1	hyper
CUBN	hyper
CUEDC1	hyper
CUX2	hypo
CWF19L1	hypo
CX3CL1	hyper
CXADR	hyper
CXCL12	hypo
CXCR2	hyper
CXXC1	hypo
CXXC5	hyper
CYB5A	hyper
CYB5R2	hyper
CYBA	hypo
CYC1	hyper
CYP11A1	hypo
CYP1A1	hypo
CYP1B1	hypo
CYP26A1	hyper
CYP26C1	hyper
CYP2J2	hypo
CYP46A1	hypo
CYP4F22	hyper
CYP4V2	hyper
CYSLTR2	hyper
CYTH2	hyper
CYTL1	hyper
CYTSB	hypo
D4S234E	hyper
DAAM2	hypo
DAB1	hypo
DACH1	hyper
DACT1	hyper
DACT2	hyper
DAGLB	hyper
DAND5	hypo
DAO	hypo
DAP	hyper
DAPK3	hyper
DAPL1	hypo
DARS	hypo
DAZAP2	hypo
DBP	hyper
DBX1	hyper
DCAF12	hypo
DCAF17	hypo
DCAF7	hyper
DCAKD	hyper
DCC	hyper
DCHS2	hyper
DCLK1	hyper
DCLK2	hyper
DCLRE1C	hypo
DCP1B	hypo

DCST1	hyper
DCTN1	hyper
DCTN4	hypo
DCUN1D5	hypo
DDC	hypo
DDHD1	hypo
DDIT4	hypo
DDIT4L	hyper
DDN	hyper
DDO	hyper
DDX18	hypo
DDX20	hyper
DDX21	hypo
DDX39	hypo
DDX43	hyper
DDX46	hyper
DDX60	hypo
DECR2	hyper
DEDD2	hypo
DEFB121	hypo
DEGS2	hypo
DENND1B	hyper
DENND3	hyper
DENND4B	hypo
DENND5A	hypo
DEPDC4	hyper
DEPDC7	hyper
DERA	hypo
DERL1	hypo
DET1	hypo
DFFB	hypo
DFNB31	hyper
DGCR14	hyper
DGCR2	hyper
DGCR5	hyper
DGCR6	hypo
DGKG	hyper
DGKI	hyper
DGKQ	hypo
DGKZ	hyper
DHRS3	hypo
DHRS7	hyper
DHX30	hyper
DHX33	hypo
DHX38	hyper
DHX40	hyper
DHX9	hypo
DIAPH1	hypo
DICER1	hyper
DIO1	hyper
DIP2B	hypo
DIRAS2	hyper
DIRC2	hyper
DIRC3	hypo
DKK1	hyper
DKK2	hyper
DKK3	hyper
DLC1	hypo

DLG1	hypo
DLG2	hyper
DLGAP3	hypo
DLK1	hyper
DLL3	hyper
DLST	hypo
DLX1	hyper
DLX4	hyper
DLX6AS	hyper
DMKN	hypo
DMP1	hyper
DMPK	hyper
DMRT2	hyper
DMRTA2	hyper
DMWD	hyper
DNAH1	hyper
DNAH5	hypo
DNAH8	hyper
DNAH9	hypo
DNAJB13	hypo
DNAJB14	hypo
DNAJB6	hyper
DNAJC10	hyper
DNAJC13	hyper
DNAJC15	hyper
DNAJC2	hyper
DNAJC27	hypo
DNAJC28	hypo
DNAJC3	hypo
DNAL4	hyper
DNASE1L2	hyper
DNER	hyper
DNM3	hyper
DNMT3A	hypo
DOCK3	hypo
DOK2	hypo
DOPEY1	hypo
DPCR1	hypo
DPEP1	hypo
DPF3	hyper
DPP10	hyper
DPP8	hypo
DPPA5	hyper
DPY19L1	hyper
DPYS	hyper
DPYSL2	hyper
DPYSL4	hyper
DPYSL5	hyper
DRD1	hyper
DRD2	hyper
DRD4	hyper
DRG1	hypo
DSC2	hyper
DSCAM	hyper
DSCR6	hyper
DSEL	hyper
DSTYK	hyper
DTNB	hyper

DTX1	hyper
DUOX1	hyper
DUOX2	hypo
DUOXA2	hyper
DUS3L	hyper
DUSP11	hyper
DUSP16	hyper
DUSP27	hypo
DUSP5	hypo
DYNC1I2	hypo
DYNC1LI1	hypo
DYNC1LI2	hypo
DYNC2LI1	hypo
DYNLRB1	hyper
DYNLT1	hypo
DYRK2	hyper
DYSF	hyper
DYX1C1	hyper
DZIP1	hypo
DZIP1L	hyper
E2F6	hyper
E2F8	hyper
E4F1	hyper
EBAG9	hypo
EBF2	hyper
EBPL	hypo
ECE1	hyper
ECE2	hyper
ECEL1	hyper
ECH1	hyper
ECHDC1	hyper
ECHS1	hypo
ECM1	hypo
ECT2L	hypo
EDAR	hypo
EDARADD	hyper
EDN1	hyper
EDN2	hypo
EDN3	hyper
EDNRB	hyper
EEF1DP3	hypo
EEF2	hypo
EFCAB1	hyper
EFCAB10	hyper
EFCAB4A	hyper
EFCAB5	hyper
EFCAB6	hypo
EFEMP1	hyper
EFHB	hypo
EFNA2	hypo
EFNA3	hyper
EFNA4	hypo
EFNB2	hypo
EGFL8	hyper
EGFLAM	hypo
EGFR	hyper
EGLN3	hyper
EGR3	hyper

EHBP1	hypo
EHBP1L1	hyper
EHD1	hypo
EHD4	hyper
EHMT1	hyper
EHMT2	hyper
EIF2AK3	hypo
EIF2B5	hypo
EIF2C1	hypo
EIF2C2	hyper
EIF2C4	hypo
EIF4E	hypo
EIF4E1B	hyper
EIF4ENIF1	hyper
EIF4G3	hyper
EIF5A	hyper
EIF5A2	hyper
ELAVL3	hyper
ELF1	hyper
ELFN2	hypo
ELOVL5	hyper
ELP3	hypo
ELTD1	hyper
EMB	hypo
EMID2	hypo
EMILIN2	hypo
EMILIN3	hyper
EML4	hyper
EMR1	hyper
EMX1	hypo
EMX2	hyper
EN2	hyper
ENHO	hypo
ENOX1	hypo
ENTPD4	hypo
ENTPD6	hyper
EOMES	hypo
EP400	hypo
EP400NL	hyper
EPB41L4B	hypo
EPHA10	hyper
EPHA4	hypo
EPHA5	hyper
EPHA8	hypo
EPHB2	hypo
EPS15	hypo
EPX	hypo
ERC1	hyper
ERC2	hypo
ERCC2	hyper
ERCC5	hypo
ERCC6	hypo
ERGIC1	hypo
ERGIC2	hypo
ERI1	hypo
ERI2	hyper
ERMAP	hypo
ERN2	hyper

ESAM	hyper
ESRRB	hypo
ESRRG	hyper
ESYT2	hypo
ESYT3	hyper
ETNK1	hyper
ETS1	hypo
ETV1	hyper
ETV4	hypo
ETV6	hyper
ETV7	hypo
EVI5L	hypo
EVX1	hyper
EVX2	hyper
EWSR1	hypo
EXD2	hypo
EXD3	hyper
EXO1	hypo
EXOC3	hyper
EXOC3L2	hypo
EXOC6B	hyper
EXOSC10	hypo
EXTL3	hyper
EYS	hypo
EZR	hypo
F2RL3	hypo
FA2H	hyper
FAF2	hypo
FAIM	hypo
FAM101B	hyper
FAM110C	hyper
FAM111A	hypo
FAM111B	hypo
FAM113A	hypo
FAM114A2	hyper
FAM116A	hypo
FAM117B	hyper
FAM118B	hyper
FAM120B	hyper
FAM123A	hyper
FAM123C	hyper
FAM124A	hyper
FAM129B	hyper
FAM13A	hyper
FAM13AOS	hyper
FAM149A	hypo
FAM150A	hypo
FAM161A	hypo
FAM163A	hypo
FAM166B	hypo
FAM168A	hyper
FAM168B	hypo
FAM172A	hypo
FAM173B	hypo
FAM176A	hypo
FAM176B	hyper
FAM178A	hypo
FAM181B	hyper

FAM184A	hyper
FAM184B	hyper
FAM188B	hypo
FAM189A1	hypo
FAM192A	hypo
FAM196A	hyper
FAM196B	hypo
FAM19A2	hypo
FAM19A5	hypo
FAM20C	hypo
FAM26F	hypo
FAM38A	hypo
FAM38B	hypo
FAM40A	hyper
FAM46B	hypo
FAM49A	hyper
FAM53A	hyper
FAM59A	hypo
FAM5B	hyper
FAM5C	hyper
FAM60A	hypo
FAM63A	hyper
FAM64A	hyper
FAM65A	hypo
FAM65B	hyper
FAM71E2	hypo
FAM76A	hyper
FAM76B	hypo
FAM82A2	hypo
FAM83A	hyper
FAM83E	hypo
FAM84B	hyper
FAM8A1	hyper
FAM91A1	hypo
FAM92A1	hyper
FAM96B	hypo
FAM98A	hypo
FAM98C	hypo
FANCC	hypo
FANCF	hypo
FARP1	hyper
FARS2	hypo
FARSA	hypo
FARSB	hypo
FASLG	hypo
FASN	hyper
FAT1	hyper
FAU	hyper
FBLL1	hyper
FBP1	hyper
FBXL15	hyper
FBXL16	hyper
FBXL17	hypo
FBXL19	hyper
FBXL21	hyper
FBXL22	hypo
FBXL4	hyper
FBXL6	hyper

FBXL7	hyper
FBXO18	hyper
FBXO22OS	hypo
FBXO3	hypo
FBXO31	hyper
FBXO34	hyper
FBXO39	hyper
FBXO41	hyper
FBXO46	hyper
FBXO7	hypo
FBXW8	hypo
FCGBP	hyper
FCGR3A	hyper
FCHO1	hypo
FCN1	hyper
FCN3	hyper
FCRL1	hypo
FCRL6	hypo
FCRLA	hypo
FDFT1	hypo
FDPS	hyper
FERD3L	hyper
FERMT3	hyper
FEV	hypo
FEZ1	hyper
FEZ2	hyper
FGA	hypo
FGF1	hypo
FGF12	hyper
FGF18	hypo
FGF19	hyper
FGF3	hyper
FGF9	hypo
FGFR3	hyper
FGR	hyper
FHDC1	hyper
FHOD3	hypo
FIBCD1	hypo
FIBIN	hyper
FIG4	hyper
FIGLA	hyper
FIGN	hypo
FIGNL2	hyper
FILIP1	hyper
FIS1	hyper
FKBP14	hypo
FKBP2	hypo
FKBP5	hyper
FKBP9	hypo
FKSG83	hyper
FLI1	hypo
FLII	hyper
FLJ10357	hyper
FLJ13197	hyper
FLJ20184	hypo
FLJ23834	hyper
FLJ26850	hyper
FLJ36000	hyper

FLJ36031	hypo
FLJ37453	hypo
FLJ39582	hypo
FLJ42289	hypo
FLJ42875	hyper
FLJ43663	hypo
FLJ45983	hypo
FLJ90757	hyper
FLNB	hypo
FLNC	hypo
FLOT1	hypo
FLRT2	hyper
FLT1	hyper
FLT4	hyper
FLVCR2	hypo
FMNL2	hypo
FMO1	hypo
FNBP1L	hyper
FNTB	hypo
FOLR4	hyper
FOXA3	hyper
FOXB1	hypo
FOXC1	hyper
FOXI1	hypo
FOXK1	hyper
FOXK2	hypo
FOXL1	hypo
FOXM1	hypo
FOXN4	hypo
FOXP1	hypo
FOXP4	hypo
FOXQ1	hyper
FPR3	hyper
FRAS1	hyper
FREM1	hypo
FREM3	hypo
FREQ	hypo
FRG1	hyper
FRMD4A	hyper
FSCN1	hypo
FSCN2	hyper
FSD1	hyper
FSHR	hyper
FSIP1	hypo
FSTL1	hyper
FSTL5	hyper
FTCD	hypo
FUBP1	hyper
FUT5	hypo
FXR2	hyper
FXYD4	hypo
FYN	hyper
FYTTD1	hyper
FZD1	hypo
FZD10	hyper
FZD6	hyper
G6PC	hyper
G6PC3	hypo

GAB2	hypo
GABARAPL1	hypo
GABARAPL2	hyper
GABRA2	hyper
GABRB1	hypo
GABRB2	hyper
GABRD	hyper
GABRG2	hyper
GADD45B	hypo
GALK2	hypo
GALNS	hypo
GALNT10	hyper
GALNT11	hypo
GALNT9	hyper
GALNTL5	hyper
GALR2	hyper
GAP43	hypo
GAPDH	hypo
GAR1	hypo
GARS	hyper
GAS2L1	hyper
GAS7	hypo
GAS8	hyper
GATA2	hyper
GATA4	hyper
GATA6	hyper
GBAP1	hypo
GBAS	hypo
GBGT1	hyper
GBX1	hyper
GC	hypo
GCA	hyper
GCH1	hypo
GCKR	hyper
GCLM	hypo
GCM1	hypo
GCM2	hyper
GCOM1	hyper
GDAP1	hyper
GDF2	hypo
GDF6	hyper
GEFT	hyper
GEMIN7	hyper
GFI1	hypo
GFOD1	hypo
GFOD2	hypo
GFPT2	hypo
GFRA2	hyper
GGA3	hypo
GGPS1	hypo
GGT1	hypo
GGTA1	hyper
GHR	hyper
GHRHR	hypo
GHRLOS	hyper
GHSR	hyper
GIMAP8	hyper
GIPC1	hypo

GIPC2	hyper
GJA3	hypo
GJA5	hyper
GJB2	hypo
GJD2	hyper
GJD3	hyper
GLB1L2	hypo
GLG1	hypo
GLI1	hypo
GLI3	hypo
GLI4	hypo
GLIPR1L2	hyper
GLRA1	hyper
GLRA3	hypo
GLRB	hyper
GLRX3	hypo
GLT1D1	hyper
GLT25D2	hypo
GLYCTK	hypo
GM2A	hypo
GMDS	hyper
GMEB2	hyper
GMPPB	hyper
GNA12	hypo
GNAI2	hypo
GNAI3	hypo
GNAO1	hyper
GNAQ	hypo
GNAS	hyper
GNASAS	hypo
GNB1	hyper
GNB1L	hypo
GNG12	hypo
GNG4	hyper
GNG7	hyper
GNL1	hyper
GNL3	hyper
GNPDA2	hyper
GNRH2	hypo
GNS	hyper
GOLPH3	hyper
GOLSYN	hypo
GON4L	hyper
GOPC	hypo
GPAM	hyper
GPATCH2	hyper
GPATCH8	hyper
GPC5	hyper
GPC6	hyper
GPI	hyper
GPR107	hyper
GPR124	hyper
GPR126	hyper
GPR132	hyper
GPR156	hypo
GPR158	hypo
GPR161	hypo
GPR176	hypo

GPR177	hypo
GPR180	hypo
GPR19	hypo
GPR20	hyper
GPR39	hyper
GPR4	hyper
GPR6	hyper
GPR61	hypo
GPR62	hypo
GPR83	hyper
GPR88	hyper
GPX2	hyper
GPX5	hypo
GPX6	hyper
GPX7	hyper
GRAMD1B	hyper
GRAMD2	hyper
GRB10	hyper
GRB2	hypo
GREB1	hyper
GREM2	hyper
GRHL2	hypo
GRID1	hyper
GRID2IP	hyper
GRIK2	hyper
GRIN2C	hyper
GRIN2D	hyper
GRIP1	hypo
GRIP2	hypo
GRK1	hypo
GRM1	hyper
GRM4	hyper
GRM5	hyper
GRM6	hyper
GRP	hyper
GRPEL1	hyper
GRRP1	hyper
GRXCR1	hypo
GSC	hyper
GSDMA	hypo
GSG1L	hyper
GSK3A	hyper
GSTP1	hyper
GSTZ1	hyper
GTF2A2	hyper
GTF2E1	hypo
GTF3C4	hypo
GTF3C6	hypo
GTPBP8	hypo
GTSF1L	hypo
GUCA2B	hypo
GUCY1A2	hyper
GUCY1B3	hyper
GUK1	hypo
GYG1	hypo
GYPC	hyper
GZMM	hyper
H19	hyper

H1FOO	hypo
H2AFV	hypo
H2AFY	hypo
H2AFY2	hyper
HAGH	hyper
HAND1	hyper
HAND2	hyper
HAPLN3	hypo
HAS2AS	hyper
HAUS2	hyper
HBEGF	hypo
HBII-52-45	hyper
HBZ	hypo
HCCA2	hypo
HCG11	hypo
HCG22	hyper
HCG27	hyper
HCN2	hyper
HCN4	hyper
HDAC10	hypo
HDAC11	hypo
HDAC5	hyper
HDAC9	hyper
HDDC3	hypo
HDGF2	hyper
HDLBP	hypo
HEATR2	hyper
HELT	hyper
HEPHL1	hyper
HEPN1	hyper
HERC2	hyper
HERPUD1	hyper
HES2	hyper
HES4	hypo
HFM1	hyper
HGC6.3	hyper
HGFAC	hypo
HHAT	hyper
HHIPL1	hyper
HHIPL2	hyper
HIAT1	hypo
HIF3A	hyper
HIGD2A	hypo
HINFP	hypo
HINT1	hypo
HIP1	hyper
HIPK2	hyper
HIPK4	hypo
HIST1H2AI	hypo
HIST1H2BE	hyper
HIST1H2BK	hypo
HIST1H3H	hypo
HIST1H4E	hypo
HIST2H2AA3	hypo
HIST2H2AC	hypo
HIST3H3	hyper
HIVEP2	hyper
HK1	hypo

HLA-DOA	hyper
HLA-DOB	hypo
HLA-DPA1	hyper
HLA-DPB1	hyper
HLA-DRA	hypo
HLA-DRB1	hypo
HLA-DRB5	hyper
HLA-F	hypo
HLA-G	hyper
HLA-J	hypo
HLF	hyper
HLTF	hyper
HMGA2	hyper
HMGCLL1	hyper
HMGXB3	hyper
HMSD	hypo
HMX2	hyper
HNF1B	hyper
HNF4A	hypo
HNRNPA1	hypo
HNRNPF	hyper
HNRNPL	hypo
HNRNPR	hypo
HNRNPUL1	hyper
HNRNPUL2	hypo
HOMER1	hypo
HOOK1	hypo
HOOK2	hypo
HOPX	hyper
HOXA10	hyper
HOXA11AS	hyper
HOXA13	hyper
HOXA2	hypo
HOXA3	hyper
HOXA4	hyper
HOXA5	hyper
HOXA6	hyper
HOXA7	hyper
HOXA9	hyper
HOXC10	hypo
HOXC11	hyper
HOXC12	hyper
HOXC13	hyper
HOXC8	hypo
HOXD11	hyper
HOXD12	hyper
HPCAL4	hyper
HPGD	hypo
HPS3	hypo
HPS4	hyper
HPSE2	hyper
HRG	hyper
HRH1	hyper
HRH3	hyper
HRK	hypo
HS3ST1	hyper
HS3ST2	hyper
HS3ST6	hypo

HSCB	hypo
HSD17B12	hypo
HSD17B2	hyper
HSD17B4	hyper
HSDL2	hypo
HSF5	hyper
HSPA1B	hypo
HSPB8	hypo
HSPC072	hyper
HSPG2	hypo
HTA	hypo
HTR1A	hyper
HTR2A	hypo
HTR3A	hypo
HTRA1	hyper
HTRA2	hypo
HTT	hypo
HYAL2	hypo
HYDIN	hypo
HYI	hyper
HYLS1	hyper
IAH1	hyper
ICA1L	hypo
ICAM1	hypo
ID3	hypo
IDO2	hypo
IER3	hypo
IFFO2	hyper
IFI27	hypo
IFI30	hyper
IFNGR2	hyper
IFT140	hypo
IFT52	hypo
IFT88	hyper
IGDCC3	hyper
IGDCC4	hypo
IGF1	hyper
IGF1R	hyper
IGF2AS	hyper
IGF2BP2	hyper
IGF2R	hyper
IGFBP3	hyper
IGFBP4	hypo
IGFBP5	hyper
IGFBP7	hyper
IGFBPL1	hypo
IGLON5	hyper
IGSF10	hyper
IGSF21	hyper
IGSF22	hyper
IGSF8	hyper
IGSF9	hyper
IGSF9B	hypo
IHH	hypo
IKZF1	hypo
IL16	hyper
IL17RA	hyper
IL17RD	hyper

IL17REL	hyper
IL19	hypo
IL1B	hyper
IL1RAP	hypo
IL21R	hypo
IL27	hypo
IL6R	hyper
IL7	hyper
ILF3	hyper
ILVBL	hyper
IMMP2L	hypo
IMP3	hypo
IMPACT	hyper
IMPG1	hyper
INA	hyper
INADL	hypo
INCENP	hypo
INF2	hypo
ING1	hypo
ING2	hypo
INHBB	hyper
INHBE	hypo
INO80	hypo
INO80D	hyper
INPP4A	hyper
INPP4B	hypo
INPP5A	hyper
INSL6	hyper
INSR	hypo
INTS1	hyper
INTS12	hyper
IPPK	hypo
IQCA1	hypo
IQCB1	hypo
IQCD	hypo
IQCE	hypo
IQCJ	hypo
IQCK	hyper
IQGAP2	hyper
IQSEC1	hyper
IRAK1BP1	hypo
IRAK2	hypo
IRF2BP2	hypo
IRF5	hyper
IRF8	hypo
IRS1	hypo
IRS2	hyper
IRX4	hyper
ISG20L2	hypo
ISLR2	hyper
ISOC2	hypo
ISPD	hyper
ISY1	hypo
ITCH	hyper
ITGA1	hypo
ITGA2	hypo
ITGA2B	hyper
ITGA8	hyper

ITGB1BP1	hyper
ITGB4	hyper
ITGBL1	hyper
ITIH5	hyper
ITPA	hyper
ITPK1	hypo
ITPKB	hypo
ITPR1	hypo
ITPR2	hyper
ITPRIP	hyper
ITSN1	hypo
ITSN2	hyper
JAG1	hyper
JAG2	hypo
JAGN1	hypo
JAK3	hypo
JAM3	hyper
JARID2	hyper
JMJD1C	hyper
JMJD6	hypo
JPH2	hyper
JRK	hyper
JUP	hypo
KAT5	hypo
KATNAL2	hyper
KAZALD1	hyper
KBTBD12	hypo
KBTBD8	hypo
KCNAB1	hypo
KCNC1	hyper
KCNC2	hyper
KCND3	hyper
KCNF1	hyper
KCNG2	hyper
KCNH2	hypo
KCNH3	hyper
KCNH5	hyper
KCNH8	hyper
KCNIP1	hyper
KCNJ15	hyper
KCNJ6	hypo
KCNK10	hypo
KCNK12	hyper
KCNK13	hyper
KCNK15	hyper
KCNK2	hyper
KCNK3	hyper
KCNK7	hyper
KCNMA1	hyper
KCNMB3	hypo
KCNMB4	hyper
KCNQ1OT1	hyper
KCNQ2	hyper
KCNS1	hyper
KCNT1	hypo
KCTD1	hypo
KCTD11	hyper
KCTD12	hyper

KCTD2	hyper
KDELR2	hypo
KDELR3	hyper
KDM4A	hyper
KDR	hyper
KHDRBS1	hypo
KHDRBS3	hypo
KHNYN	hyper
KIAA0090	hypo
KIAA0146	hyper
KIAA0182	hyper
KIAA0196	hypo
KIAA0232	hyper
KIAA0317	hyper
KIAA0415	hyper
KIAA0556	hyper
KIAA0562	hyper
KIAA0649	hyper
KIAA0754	hyper
KIAA0776	hypo
KIAA0831	hyper
KIAA0895L	hyper
KIAA0907	hyper
KIAA1024	hyper
KIAA1033	hypo
KIAA1045	hypo
KIAA1109	hypo
KIAA1161	hyper
KIAA1191	hyper
KIAA1274	hypo
KIAA1328	hypo
KIAA1383	hyper
KIAA1409	hypo
KIAA1462	hypo
KIAA1467	hypo
KIAA1522	hyper
KIAA1549	hyper
KIAA1598	hyper
KIAA1644	hyper
KIAA1688	hypo
KIAA1751	hypo
KIAA1755	hyper
KIAA1804	hypo
KIAA1984	hyper
KIF12	hypo
KIF15	hypo
KIF19	hyper
KIF1A	hyper
KIF1B	hypo
KIF23	hypo
KIF25	hyper
KIF3A	hypo
KIF9	hypo
KIR2DL4	hypo
KIR3DL1	hypo
KIR3DL3	hypo
KIRREL	hypo
KIRREL3	hyper

KISS1	hyper
KIT	hyper
KLC1	hyper
KLC2	hypo
KLC3	hyper
KLC4	hypo
KLF1	hyper
KLF13	hyper
KLF6	hypo
KLF7	hyper
KLHDC8A	hypo
KLHL1	hyper
KLHL14	hyper
KLHL2	hyper
KLHL30	hyper
KLHL31	hyper
KLHL32	hyper
KLHL35	hyper
KLHL36	hyper
KLHL38	hypo
KLHL6	hypo
KLK10	hyper
KLK11	hypo
KLK15	hypo
KLRAQ1	hypo
KNG1	hypo
KPNA1	hyper
KPTN	hypo
KRCC1	hypo
KREMEN2	hyper
KRIT1	hypo
KRR1	hypo
KRT1	hypo
KRT33A	hyper
KRT5	hypo
KRT6B	hypo
KRT6C	hypo
KRT7	hyper
KRT73	hypo
KRT74	hypo
KRT78	hypo
KRT83	hypo
KRT85	hyper
KRTAP20-1	hypo
KRTAP2-1	hypo
KRTAP26-1	hyper
KRTAP3-1	hypo
KRTAP4-5	hypo
L3MBTL2	hypo
L3MBTL3	hypo
LAMB1	hyper
LAMB3	hyper
LAMC1	hypo
LAMC3	hyper
LAP3	hypo
LAPTM4A	hyper
LARP1	hyper
LASS3	hyper

LASS5	hyper
LATS2	hyper
LBXCOR1	hypo
LCLAT1	hyper
LCN10	hypo
LCN6	hypo
LCN9	hypo
LCP2	hyper
LDB2	hypo
LDHA	hypo
LDHAL6A	hyper
LDHD	hypo
LDLR	hypo
LDLRAD3	hyper
LEKR1	hypo
LEO1	hypo
LEP	hyper
LEPRE1	hyper
LEPREL1	hyper
LETM1	hyper
LETM2	hypo
LGALS3	hypo
LGALS3BP	hypo
LGI1	hyper
LGI3	hypo
LGR5	hyper
LHFPL4	hyper
LHX1	hyper
LHX2	hyper
LHX4	hyper
LHX5	hyper
LHX8	hyper
LHX9	hyper
LIAS	hypo
LILRB1	hypo
LIMCH1	hypo
LIMK1	hypo
LIMK2	hyper
LIN7B	hypo
LINGO1	hypo
LINGO3	hyper
LIPC	hypo
LIPG	hypo
LMAN1L	hyper
LMBR1	hypo
LMF1	hyper
LMLN	hypo
LMO7	hypo
LMOD2	hypo
LMTK2	hypo
LMTK3	hypo
LMX1A	hyper
LNP1	hyper
LNPEP	hyper
LOC100128023	hyper
LOC100128554	hyper
LOC100128788	hypo
LOC100130274	hyper

LOC100130522	hyper
LOC100130776	hyper
LOC100130872	hyper
LOC100132215	hyper
LOC100133091	hypo
LOC100133991	hyper
LOC100134713	hypo
LOC100268168	hyper
LOC100288797	hypo
LOC100302652	hypo
LOC100329108	hypo
LOC115110	hyper
LOC116437	hypo
LOC145814	hypo
LOC146336	hyper
LOC149620	hypo
LOC150381	hypo
LOC151174	hypo
LOC151534	hyper
LOC254312	hyper
LOC257358	hyper
LOC282997	hypo
LOC284412	hyper
LOC284688	hyper
LOC284805	hyper
LOC285375	hypo
LOC285419	hypo
LOC285501	hyper
LOC285548	hyper
LOC285550	hypo
LOC285780	hyper
LOC387647	hyper
LOC387763	hypo
LOC388428	hyper
LOC388796	hypo
LOC388946	hyper
LOC389033	hyper
LOC390595	hyper
LOC391322	hypo
LOC400752	hyper
LOC400940	hypo
LOC401052	hypo
LOC441601	hyper
LOC441666	hyper
LOC441897	hyper
LOC554202	hyper
LOC619207	hypo
LOC641518	hypo
LOC642006	hypo
LOC643406	hypo
LOC645323	hyper
LOC648691	hypo
LOC728024	hyper
LOC728392	hyper
LOC729176	hyper
LOC729991-ME	hypo
LOC732275	hyper
LOR	hyper

LOXL2	hyper
LPCAT2	hyper
LPIN1	hyper
LPIN2	hyper
LPIN3	hypo
LPP	hyper
LRCH1	hyper
LRDD	hyper
LRFN5	hyper
LRIG1	hyper
LRIT1	hypo
LRP1	hyper
LRP10	hyper
LRP2	hyper
LRRC10	hypo
LRRC10B	hyper
LRRC14	hypo
LRRC14B	hyper
LRRC15	hypo
LRRC2	hypo
LRRC20	hyper
LRRC23	hypo
LRRC27	hyper
LRRC33	hypo
LRRC43	hyper
LRRC48	hypo
LRRC4B	hypo
LRRC56	hypo
LRRC8C	hypo
LRRCC1	hypo
LRRFIP2	hypo
LRRIQ4	hyper
LRRN2	hypo
LRTM2	hyper
LSG1	hypo
LSM5	hyper
LSR	hypo
LTBP1	hyper
LTBP2	hyper
LTBP4	hypo
LUC7L2	hyper
LUC7L3	hypo
LY6E	hypo
LY6G5C	hypo
LY6G6C	hypo
LY6G6D	hypo
LY6G6F	hypo
LY6K	hyper
LY9	hypo
LYPD4	hyper
LYPD6B	hyper
LYPLA2	hyper
LYRM2	hyper
LYST	hyper
LYZ	hyper
LZTS1	hyper
MACF1	hyper
MACROD1	hyper

MACROD2	hyper
MAD2L1	hypo
MAD2L1BP	hypo
MADCAM1	hyper
MAEA	hypo
MAFB	hyper
MAGEF1	hyper
MAGEL2	hyper
MAGI2	hypo
MAGI3	hypo
MAGOH	hypo
MALT1	hypo
MAML3	hyper
MAMSTR	hypo
MAN2A1	hyper
MAN2B1	hypo
MANBA	hypo
MAP1D	hyper
MAP1LC3A	hypo
MAP2	hyper
MAP2K3	hypo
MAP2K4	hyper
MAP2K5	hyper
MAP2K7	hypo
MAP3K1	hypo
MAP3K11	hypo
MAP3K12	hyper
MAP3K5	hypo
MAP3K7	hypo
MAP3K7IP1	hypo
MAP3K7IP2	hyper
MAP4K4	hyper
MAP6D1	hyper
MAPK12	hypo
MAPK14	hypo
MAPK8	hyper
MARCO	hyper
MARK4	hyper
MARS2	hypo
MASP2	hypo
MAST1	hyper
MAST3	hyper
MAST4	hypo
MATK	hypo
MATR3	hyper
MAZ	hypo
MB	hypo
MBD3L1	hypo
MBD6	hyper
MBIP	hypo
MBNL2	hyper
MBP	hyper
MC1R	hyper
MCF2L2	hypo
MCFD2	hypo
MCL1	hyper
MCM10	hyper
MCM7	hypo

MCM8	hypo
MCOLN1	hypo
MCOLN3	hypo
MCPH1	hyper
MDH1B	hyper
MDM1	hypo
MEAF6	hypo
MECOM	hyper
MED1	hypo
MED12L	hyper
MED18	hypo
MED19	hypo
MED20	hypo
MED24	hyper
MED28	hypo
MED31	hypo
MEF2A	hyper
MEF2C	hyper
MEFV	hyper
MEG3	hyper
MEGF11	hyper
MEGF8	hypo
MEIG1	hypo
MEIS1	hyper
MEIS2	hyper
MESDC1	hypo
MET	hypo
METRN	hypo
METRNL	hypo
METT10D	hypo
METTL11B	hyper
METTL13	hyper
METTL7B	hypo
MEX3B	hyper
MEX3D	hyper
MFAP3L	hypo
MFF	hypo
MFI2	hypo
MFN1	hypo
MFRP	hypo
MFSD3	hypo
MFSD4	hypo
MFSD7	hyper
MFSD8	hypo
MFSD9	hyper
MGA	hyper
MGAM	hypo
MGC23284	hyper
MGC2752	hypo
MGLL	hyper
MGST2	hyper
MGST3	hyper
MIAT	hypo
MICAL3	hyper
MICB	hypo
MIDN	hyper
MIER2	hyper
MIOX	hypo

MIR1253	hyper
MIR1275	hypo
MIR128-2	hyper
MIR1323	hyper
MIR181B1	hypo
MIR190B	hypo
MIR200A	hypo
MIR29B1	hypo
MIR29C	hyper
MIR30C2	hypo
MIR330	hypo
MIR33B	hyper
MIR483	hypo
MIR487A	hypo
MIR493	hypo
MIR520A	hypo
MIR548H4	hypo
MIR548N	hyper
MIR563	hyper
MIR574	hyper
MIR596	hyper
MIR629	hypo
MIR638	hypo
MIR663	hyper
MIR758	hypo
MIR769	hypo
MIR874	hypo
MIR942	hypo
MIS12	hypo
MKI67	hypo
MKL2	hypo
MKLN1	hyper
MKRN1	hypo
MKS1	hypo
MLC1	hyper
MLF2	hyper
MLH1	hypo
MLL3	hyper
MLL4	hypo
MLLT1	hypo
MLN	hypo
MLPH	hypo
MLXIPL	hypo
MMADHC	hypo
MMD2	hyper
MME	hyper
MMEL1	hyper
MMP14	hypo
MMP21	hyper
MMP25	hypo
MMP26	hyper
MMP9	hyper
MMRN1	hyper
MN1	hyper
MND1	hypo
MNT	hypo
MNX1	hyper
MOBKL2A	hyper

MOCS2	hypo
MOG	hyper
MOGAT3	hypo
MOGS	hypo
MON1A	hyper
MON2	hypo
MORC3	hypo
MORN1	hypo
MOSC1	hypo
MOSC2	hypo
MOV10	hyper
MPP2	hyper
MPP3	hyper
MPP5	hyper
MPPE1	hypo
MPRIP	hyper
MRC2	hypo
MRE11A	hyper
MRGPRE	hyper
MRGPRF	hyper
MRGPRX2	hypo
MRI1	hypo
MRM1	hyper
MRPL14	hypo
MRPL15	hypo
MRPL17	hypo
MRPL30	hypo
MRPL36	hypo
MRPL43	hypo
MRPL48	hypo
MRPL54	hypo
MRPL55	hypo
MRPS10	hyper
MRPS22	hypo
MRPS24	hypo
MRPS27	hyper
MRPS36	hypo
MRTO4	hypo
MS4A15	hypo
MS4A6E	hypo
MSH3	hyper
MSI1	hyper
MSL2	hyper
MSLN	hypo
MSLNL	hypo
MSRB3	hypo
MST1R	hyper
MT1B	hypo
MT1E	hypo
MTF2	hyper
MTFR1	hypo
MTHFSD	hyper
MTIF3	hyper
MTMR12	hyper
MTMR15	hypo
MTMR7	hyper
MTMR9L	hyper
MTOR	hypo

MTPAP	hypo
MTR	hypo
MTSS1	hypo
MTUS1	hyper
MTUS2	hyper
MUC1	hypo
MUC12	hyper
MUC13	hypo
MUC15	hyper
MUC16	hypo
MUC2	hypo
MUDENG	hypo
MX1	hypo
MXD1	hypo
MXD4	hypo
MYADM	hyper
MYADML2	hypo
MYBBP1A	hypo
MYBPC1	hyper
MYCBP	hypo
MYCBPAP	hyper
MYH10	hypo
MYH16	hypo
MYH3	hyper
MYH4	hypo
MYH6	hypo
MYH8	hyper
MYH9	hyper
MYLK4	hypo
MYO15A	hypo
MYO15B	hyper
MYO18A	hyper
MYO1D	hypo
MYO1G	hyper
MYO9A	hyper
MYO9B	hypo
MYOC	hypo
MYOD1	hyper
MYOM3	hyper
MYST4	hypo
MZF1	hyper
N6AMT2	hyper
NAA15	hypo
NAA38	hypo
NAALADL2	hyper
NAB1	hypo
NAB2	hypo
NACA2	hypo
NACC1	hypo
NADSYN1	hypo
NAF1	hyper
NAGA	hypo
NAMPT	hypo
NANOG	hypo
NAPB	hypo
NAT15	hyper
NAV1	hyper
NBEA	hyper

NBEAL2	hypo
NBL1	hypo
NCAM1	hypo
NCAPG	hypo
NCAPH	hypo
NCF2	hypo
NCK2	hyper
NCKAP5	hyper
NCLN	hypo
NCOR2	hyper
NCRNA00092	hyper
NCRNA00111	hypo
NCRNA00174	hypo
NCRNA00175	hypo
NCSTN	hypo
NDC80	hypo
NDEL1	hyper
NDFIP2	hyper
NDN	hypo
NDRG3	hyper
NDUFA10	hyper
NDUFA12	hypo
NDUFA13	hypo
NDUFA4	hypo
NDUFA8	hyper
NDUFB4	hypo
NDUFB7	hypo
NDUFB8	hypo
NDUFS4	hyper
NDUFS6	hypo
NDUFS7	hyper
NDUFV2	hypo
NEAT1	hypo
NEB	hypo
NECAP2	hypo
NEDD4	hypo
NEDD9	hyper
NEFH	hyper
NEK10	hyper
NEK3	hypo
NEK5	hyper
NEK6	hyper
NEK8	hypo
NELF	hyper
NELL1	hyper
NEU1	hyper
NEUROD4	hyper
NEUROG1	hyper
NEUROG2	hypo
NEUROG3	hyper
NFAM1	hypo
NFATC2IP	hyper
NFATC4	hyper
NFE2L3	hypo
NFIB	hyper
NFIC	hyper
NFIX	hypo
NFKB2	hypo

NFXL1	hyper
NFYC	hyper
NGEF	hypo
NGFR	hypo
NHEJ1	hyper
NHLRC1	hyper
NHP2	hypo
NHSL1	hyper
NICN1	hyper
NID2	hyper
NIN	hyper
NIPA1	hypo
NIPAL2	hyper
NIPBL	hypo
NIPSNAP3B	hyper
NKAIN1	hypo
NKAIN4	hypo
NKAPL	hyper
NKX2-2	hyper
NKX2-3	hyper
NKX2-8	hyper
NKX3-2	hyper
NKX6-1	hyper
NLGN1	hyper
NLGN2	hyper
NLK	hypo
NMBR	hyper
NME2	hypo
NME6	hypo
NME7	hypo
NMNAT1	hyper
NMNAT3	hyper
NMT2	hyper
NMUR2	hypo
NOD2	hyper
NOL10	hypo
NOL4	hyper
NOP16	hypo
NOS1	hyper
NOSTRIN	hyper
NOTCH3	hyper
NPAS3	hyper
NPAS4	hyper
NPC2	hypo
NPDC1	hypo
NPFF	hyper
NPHP1	hyper
NPHS2	hypo
NPM1	hypo
NPR3	hyper
NPTN	hyper
NPTX1	hypo
NPTX2	hyper
NPVF	hyper
NPY	hypo
NR2E1	hyper
NR2F1	hypo
NR2F6	hyper

NR3C2	hyper
NR4A2	hyper
NR5A1	hypo
NR6A1	hyper
NRAP	hyper
NRARP	hyper
NRBP2	hypo
NRCAM	hypo
NRF1	hypo
NRG1	hyper
NRGN	hyper
NRP2	hypo
NRSN1	hyper
NRXN3	hyper
NSL1	hypo
NSMCE1	hyper
NSUN5	hyper
NT5C1B	hyper
NT5C3L	hypo
NTAN1	hyper
NTF3	hyper
NTNG1	hyper
NTNG2	hyper
NTSR2	hyper
NUB1	hypo
NUDCD2	hyper
NUDT12	hyper
NUDT18	hypo
NUDT22	hypo
NUMA1	hyper
NUMBL	hypo
NUP153	hypo
NUP155	hypo
NUP37	hypo
NXPH1	hyper
NXPH2	hyper
NXT1	hypo
NYNRIN	hypo
OAF	hyper
OBFC1	hypo
OBSL1	hyper
ODC1	hyper
ODF2	hyper
ODZ2	hypo
ODZ3	hyper
OGDHL	hyper
OGFOD2	hypo
OGFR	hypo
OLFM1	hyper
OLFM2	hyper
OLIG2	hyper
ONECUT1	hyper
ONECUT3	hyper
OPALIN	hypo
OPRL1	hypo
OPTN	hypo
OR10AD1	hyper
OR10C1	hyper

OR11A1	hypo
OR11H6	hypo
OR11L1	hypo
OR1G1	hypo
OR2A14	hypo
OR2L1P	hypo
OR2Z1	hypo
OR4C6	hypo
OR4D2	hyper
OR4F6	hyper
OR52E2	hypo
OR52I2	hypo
OR5AS1	hyper
OR5AU1	hypo
OR5K3	hyper
OR5P3	hypo
OR6B1	hyper
OR7G1	hypo
OR8D1	hyper
OR8U8	hypo
ORAOV1	hyper
ORC4L	hypo
ORC5L	hypo
OSBP	hyper
OSBP2	hypo
OSBPL10	hyper
OSBPL5	hyper
OSGIN1	hyper
OSMR	hyper
OSR1	hyper
OSR2	hyper
OSTalpha	hypo
OSTBETA	hyper
OSTC	hyper
OSTM1	hyper
OTOF	hypo
OTP	hyper
OTUD4	hypo
OTUD6B	hypo
OTX1	hyper
OVCH2	hypo
OVGP1	hypo
OXER1	hyper
OXGR1	hyper
P2RX4	hyper
P2RX5	hypo
P2RY6	hypo
P4HA2	hypo
P4HA3	hyper
PACS1	hypo
PACSIN1	hyper
PADI4	hyper
PAF1	hypo
PAFAH1B1	hypo
PAG1	hyper
PAIP2B	hypo
PAK4	hyper
PAK6	hyper

PALLD	hyper
PANK1	hypo
PANK2	hypo
PANK4	hyper
PAOX	hypo
PAPPA2	hyper
PAPSS1	hypo
PAQR5	hyper
PAQR8	hyper
PARD3B	hyper
PARN	hyper
PARP16	hyper
PARP2	hypo
PARP4	hypo
PAR-SN	hyper
PARVG	hyper
PATE3	hypo
PAX1	hypo
PAX2	hyper
PAX6	hyper
PAX9	hyper
PBX1	hyper
PCBP2	hypo
PCCB	hypo
PCDH17	hyper
PCDH18	hyper
PCDH24	hyper
PCDH9	hypo
PCDHA1	hyper
PCDHA2	hyper
PCDHA7	hypo
PCDHA9	hyper
PCDHB1	hyper
PCDHB14	hyper
PCDHB15	hyper
PCDHB17	hyper
PCDHB18	hyper
PCDHB19P	hyper
PCDHB2	hyper
PCDHB4	hyper
PCDHB5	hyper
PCDHGA1	hyper
PCDHGA2	hyper
PCDHGB4	hyper
PCGF2	hypo
PCGF3	hyper
PCIF1	hypo
PCK1	hypo
PCMTD2	hypo
PCNXL2	hyper
PCOLCE2	hyper
PCSK1	hyper
PCSK6	hypo
PCSK9	hyper
PCYOX1	hyper
PCYT1A	hypo
PDAP1	hypo
PDCD5	hyper

PDCD7	hyper
PDE12	hypo
PDE1A	hyper
PDE4A	hypo
PDE4DIP	hypo
PDE6A	hypo
PDE6B	hyper
PDE6D	hypo
PDE6G	hypo
PDE7B	hyper
PDE8A	hypo
PDE9A	hyper
PDGFA	hypo
PDGFD	hyper
PDHA2	hypo
PDIA4	hyper
PDIA5	hyper
PDIA6	hypo
PDLIM1	hyper
PDLIM3	hyper
PDP1	hypo
PDPK1	hyper
PDPN	hyper
PDSS1	hyper
PDXP	hyper
PEBP4	hypo
PEG3	hyper
PELI1	hypo
PELI2	hyper
PENK	hyper
PER2	hypo
PER3	hyper
PEX11A	hyper
PEX14	hypo
PEX5	hypo
PFDN6	hypo
PFN3	hypo
PGAM1	hypo
PGAM2	hyper
PGAP3	hyper
PGBD5	hyper
PGGT1B	hypo
PGPEP1	hyper
PGS1	hypo
PHACTR3	hyper
PHF12	hypo
PHF15	hypo
PHF20L1	hyper
PHF21A	hypo
PHF21B	hyper
PHF7	hyper
PHLDA2	hypo
PHLDB1	hypo
PHLPP2	hypo
PHOX2B	hypo
PHRF1	hyper
PHTF2	hypo
PHYH	hypo

PHYHIPL	hyper
PI15	hypo
PI4K2B	hypo
PIAS1	hyper
PIGB	hypo
PIGO	hypo
PIGY	hypo
PIGZ	hypo
PIK3AP1	hypo
PIK3IP1	hyper
PIM3	hypo
PINK1	hyper
PIP4K2A	hyper
PIP5K1C	hyper
PIP5KL1	hypo
PITPNM2	hyper
PIWIL1	hyper
PKD1	hyper
PKD1L2	hyper
PKDCC	hypo
PKIB	hyper
PKIG	hyper
PKLR	hyper
PKNOX1	hypo
PLA2G15	hypo
PLA2G1B	hypo
PLA2G2E	hypo
PLA2G2F	hypo
PLA2G3	hypo
PLA2G6	hypo
PLAA	hypo
PLAC2	hyper
PLAC8	hyper
PLAGL1	hypo
PLAT	hypo
PLB1	hypo
PLCB2	hyper
PLCB4	hyper
PLCG2	hyper
PLCXD2	hypo
PLEC1	hypo
PLEKHA2	hyper
PLEKHA4	hyper
PLEKHA6	hypo
PLEKHB1	hyper
PLEKHF2	hypo
PLEKHG1	hyper
PLEKHG2	hypo
PLEKHG4	hyper
PLEKHH2	hyper
PLEKHH3	hypo
PLEKHN1	hypo
PLIN4	hypo
PLLP	hyper
PLOD2	hyper
PLOD3	hypo
PLSCR1	hyper
PLSCR2	hyper

PLSCR3	hypo
PLSCR4	hypo
PLVAP	hyper
PLXNC1	hyper
PM20D2	hypo
PMEPA1	hypo
PML	hyper
PMM2	hypo
PMPCA	hyper
PMPCB	hyper
PMS1	hyper
PMVK	hypo
PNKP	hypo
PNLDC1	hyper
PNLIP	hyper
PNMA1	hypo
PNOC	hyper
PNPLA7	hypo
PNPT1	hypo
PODN	hypo
PODXL2	hyper
POLD2	hypo
POLG2	hypo
POLR1B	hyper
POLR1D	hyper
POLR1E	hypo
POLR2C	hypo
POLR2G	hypo
POLR2I	hypo
POLR3G	hypo
POLR3H	hypo
POLS	hyper
POMGNT1	hyper
PON2	hyper
PON3	hyper
POP1	hyper
POPDC2	hypo
POSTN	hypo
POTEC	hyper
POU2F2	hyper
POU3F1	hypo
POU4F1	hyper
POU4F2	hyper
POU4F3	hyper
POU5F1	hyper
POU6F2	hyper
PPAPDC1A	hyper
PPARD	hypo
PPCDC	hypo
PPFIA1	hypo
PPFIA3	hypo
PPHLN1	hypo
PPIG	hypo
PPIH	hypo
PPIL6	hyper
PPL	hypo
PPM1A	hypo
PPM1J	hypo

PPM1K	hypo
PPM1L	hypo
PPME1	hyper
PPP1R11	hyper
PPP1R13L	hypo
PPP1R14B	hypo
PPP1R16B	hypo
PPP1R2	hyper
PPP1R2P3	hyper
PPP1R3B	hypo
PPP1R3C	hyper
PPP1R3G	hyper
PPP1R7	hyper
PPP1R9B	hypo
PPP2R1B	hyper
PPP2R2B	hyper
PPP2R2D	hyper
PPP2R4	hyper
PPP2R5B	hypo
PPP4R1	hyper
PPP4R2	hypo
PPRC1	hypo
PPT1	hypo
PRAM1	hyper
PRAME	hypo
PRCD	hypo
PRDM1	hypo
PRDM11	hyper
PRDM13	hyper
PRDM15	hyper
PRDM5	hypo
PRDM6	hypo
PRDM8	hypo
PREPL	hypo
PREX1	hyper
PRG2	hypo
PRHOXNB	hyper
PRICKLE1	hyper
PRICKLE2	hypo
PRICKLE4	hypo
PRKAB2	hypo
PRKACA	hypo
PRKAG3	hypo
PRKAR1A	hypo
PRKCB	hyper
PRKCD	hyper
PRKCG	hyper
PRKCI	hyper
PRKD2	hyper
PRKG2	hypo
PRLHR	hyper
PRMT1	hypo
PRMT3	hypo
PRMT7	hyper
PROKR1	hypo
PROX1	hyper
PROZ	hypo
PRPF38B	hyper

PRPF8	hyper
PRPH	hyper
PRPH2	hypo
PRPSAP1	hypo
PRR14	hyper
PRR16	hyper
PRR18	hyper
PRR3	hypo
PRRC1	hyper
PRRT3	hyper
PRSS35	hypo
PRTFDC1	hypo
PRTN3	hyper
PSD	hyper
PSKH2	hyper
PSMA5	hypo
PSMA7	hyper
PSMA8	hyper
PSMB2	hypo
PSMB3	hypo
PSMB8	hypo
PSMB9	hypo
PSMD7	hypo
PSME4	hypo
PSMF1	hyper
PSORS1C1	hypo
PSTPIP1	hyper
PTCH1	hypo
PTDSS2	hyper
PTER	hyper
PTGDR	hyper
PTGER2	hyper
PTGER3	hyper
PTGIS	hyper
PTH1R	hypo
PTH2	hyper
PTH2R	hyper
PTHLH	hyper
PTK2B	hypo
PTMA	hypo
PTPLAD1	hypo
PTPMT1	hyper
PTPN11	hyper
PTPN13	hyper
PTPN18	hyper
PTPN3	hyper
PTPRC	hypo
PTPRE	hypo
PTPRF	hypo
PTPRG	hyper
PTPRO	hyper
PTPRS	hyper
PTPRU	hyper
PTRF	hyper
PTTG1	hypo
PTTG1IP	hypo
PUM1	hypo
PURA	hypo

PURG	hyper
PVRL4	hyper
PVT1	hyper
PWWP2A	hypo
PXDNL	hyper
PXMP3	hypo
PXN	hyper
PXT1	hypo
PYCR1	hyper
PYCR2	hypo
PYCRL	hypo
PYGO1	hyper
PYROXD1	hypo
QSOX1	hyper
QSOX2	hypo
RAB11FIP3	hypo
RAB13	hyper
RAB18	hypo
RAB21	hypo
RAB22A	hypo
RAB27A	hypo
RAB2B	hypo
RAB30	hypo
RAB36	hyper
RAB37	hyper
RAB38	hyper
RAB40B	hypo
RAB4A	hypo
RAB8A	hypo
RAB9P1	hyper
RABEPK	hypo
RABIF	hyper
RABL4	hypo
RAD18	hypo
RAD23B	hypo
RAD50	hyper
RAD51AP1	hyper
RAD51L1	hypo
RAD52	hypo
RAD9A	hypo
RAET1K	hypo
RAI1	hyper
RALGPS1	hypo
RAP1A	hypo
RAP1GAP2	hyper
RAP2B	hypo
RAPGEF5	hyper
RAPGEF6	hypo
RARG	hypo
RARRES2	hyper
RARRES3	hypo
RARS	hyper
RASAL2	hyper
RASD1	hypo
RASGRF1	hyper
RASGRP2	hyper
RASIP1	hypo
RASL10B	hyper

RASL11B	hyper
RASSF1	hyper
RASSF10	hypo
RASSF2	hypo
RASSF4	hyper
RASSF6	hyper
RAX2	hyper
RBBP6	hyper
RBM14	hypo
RBM15B	hypo
RBM19	hypo
RBM27	hyper
RBM33	hypo
RBM38	hypo
RBM43	hypo
RBM47	hypo
RBM6	hypo
RBMXL1	hypo
RBP1	hypo
RBP7	hyper
RBPJ	hypo
RC3H1	hyper
RC3H2	hyper
RCAN2	hyper
RCC2	hyper
RCVRN	hyper
RDH11	hyper
RDH14	hyper
RDX	hyper
REEP4	hypo
RELN	hyper
RER1	hyper
REST	hyper
REV1	hypo
REXO1	hyper
REXO2	hypo
RFESD	hypo
RFPL2	hyper
RFPL3S	hyper
RFTN1	hyper
RFWD3	hypo
RFX2	hyper
RFX4	hyper
RFX6	hyper
RFXAP	hypo
RG9MTD2	hypo
RGL3	hyper
RGP1	hypo
RGS10	hypo
RGS12	hyper
RGS17	hypo
RGS5	hyper
RGS7BP	hyper
RHBDD2	hypo
RHBDL3	hypo
RHBG	hypo
RHCG	hypo
RHOBTB2	hypo

RHOBTB3	hyper
RHOD	hyper
RHOF	hypo
RHOH	hypo
RHOT2	hyper
RIBC2	hyper
RICH2	hypo
RICTOR	hypo
RILPL1	hyper
RIMS1	hyper
RIMS2	hyper
RIMS4	hyper
RING1	hypo
RIOK2	hypo
RIPK3	hyper
RIPK4	hypo
RLTPR	hyper
RMST	hypo
RNASE1	hypo
RNASE13	hypo
RND1	hyper
RNF114	hyper
RNF126	hypo
RNF14	hypo
RNF152	hyper
RNF157	hypo
RNF186	hypo
RNF20	hyper
RNF212	hyper
RNF216L	hyper
RNF220	hyper
RNF34	hypo
RNF38	hyper
RNF4	hypo
RNF40	hypo
RNF43	hypo
RNF5P1	hypo
RNFT2	hypo
RNGTT	hypo
RNMTL1	hyper
RNPC3	hypo
RNPEP	hypo
RNU5E	hyper
ROBO1	hyper
ROBO2	hyper
ROBO3	hyper
ROR2	hyper
RORA	hypo
ROS1	hyper
RPAIN	hypo
RPL10A	hypo
RPL11	hypo
RPL13	hypo
RPL13AP20	hypo
RPL13AP5	hypo
RPL14	hyper
RPL23A	hypo
RPL24	hypo

RPL28	hypo
RPL29	hypo
RPL30	hypo
RPL35	hypo
RPL41	hypo
RPL5	hypo
RPL6	hypo
RPLP2	hypo
RPN1	hypo
RPP14	hypo
RPP30	hypo
RPRD2	hyper
RPS14	hypo
RPS18	hypo
RPS19	hypo
RPS2	hypo
RPS27A	hypo
RPS3	hypo
RPS6KA1	hyper
RPS6KC1	hypo
RPS6KL1	hyper
RPSAP52	hypo
RPUSD1	hypo
RRAGD	hyper
RREB1	hyper
RRM1	hypo
RRM2B	hypo
RRS1	hypo
RSPO2	hyper
RSRC1	hypo
RTBDN	hypo
RTDR1	hypo
RTL1	hyper
RTN3	hypo
RTN4	hypo
RTN4R	hypo
RTN4RL1	hyper
RTTN	hypo
RUFY1	hyper
RUFY3	hyper
RUNDC3A	hyper
RUNX1	hyper
RUNX1T1	hypo
RUSC1	hyper
RUVBL1	hypo
RYR1	hyper
RYR2	hypo
RYR3	hypo
S100A10	hypo
S100A13	hyper
S100B	hypo
S1PR3	hypo
S1PR5	hypo
SACM1L	hypo
SAFB	hyper
SALL3	hyper
SAMD11	hyper
SAMD12	hypo

SAMD13	hyper
SAMD14	hypo
SAMD4A	hypo
SAMD5	hyper
SAMD9L	hyper
SART3	hypo
SAV1	hyper
SBF1	hyper
SBF2	hypo
SBNO1	hyper
SCAF1	hypo
SCAND2	hyper
SCAND3	hypo
SCAP	hypo
SCARNA4	hyper
SCD	hyper
SCFD1	hypo
SCG5	hyper
SCGB1D1	hyper
SCLY	hyper
SCML4	hypo
SCN4A	hyper
SCN5A	hypo
SCNN1A	hyper
SCNN1G	hyper
SCO1	hypo
SCP2	hyper
SCRIB	hyper
SCRN1	hypo
SCRT1	hyper
SCRT2	hyper
SCT	hyper
SCTR	hypo
SCYL2	hyper
SDC1	hypo
SDC2	hypo
SDCCAG8	hyper
SDHAF1	hypo
SDR16C5	hyper
SEC14L1	hypo
SEC14L4	hypo
SEC16A	hyper
SEC22C	hypo
SEC23B	hyper
SEC24B	hyper
SEC24D	hyper
SEC63	hypo
SECISBP2L	hypo
SECTM1	hypo
SELM	hypo
SELO	hypo
SELT	hypo
SELV	hypo
SEMA3B	hyper
SEMA3C	hypo
SEMA3D	hypo
SEMA4B	hyper
SEMA5A	hyper

SEMA5B	hypo
SEMG1	hypo
SENP7	hyper
SENP8	hypo
SEPHS2	hyper
SEPN1	hyper
SEPW1	hypo
SERGEF	hyper
SERHL2	hypo
SERINC3	hypo
SERINC4	hyper
SERPINH1	hypo
SERPINI1	hypo
SESN2	hypo
SESN3	hypo
SETBP1	hypo
SETD1B	hyper
SETX	hypo
SEZ6	hyper
SEZ6L	hyper
SF1	hypo
SF3A2	hyper
SF3B2	hypo
SFMBT2	hyper
SFRP4	hyper
SFRP5	hyper
SFRS2IP	hypo
SFRS8	hyper
SFTA3	hyper
SGCB	hyper
SGCD	hyper
SGEF	hyper
SGIP1	hyper
SGK196	hyper
SGK3	hyper
SGMS1	hypo
SGSH	hyper
SGSM3	hyper
SGTA	hyper
SH2D4A	hyper
SH2D5	hyper
SH3BGRL3	hyper
SH3BP5L	hyper
SH3D19	hyper
SH3GLB1	hypo
SH3GLB2	hypo
SH3RF1	hyper
SH3TC1	hyper
SH3YL1	hyper
SHC3	hyper
SHF	hyper
SHH	hyper
SHISA3	hyper
SHISA6	hypo
SHISA7	hyper
SHISA9	hyper
SHMT2	hyper
SHOX2	hyper

SHROOM1	hypo
SIGLEC10	hyper
SIGLEC15	hyper
SIGLEC5	hypo
SIGLEC9	hyper
SIK1	hyper
SIM1	hyper
SIM2	hyper
SIN3A	hypo
SIPA1L1	hyper
SIRPB2	hyper
SIRT2	hypo
SIRT3	hypo
SIRT4	hyper
SKAP2	hypo
SKI	hypo
SKP2	hypo
SLAIN1	hypo
SLAMF8	hyper
SLC10A6	hypo
SLC12A1	hypo
SLC12A5	hyper
SLC12A7	hyper
SLC16A12	hyper
SLC16A13	hypo
SLC16A3	hyper
SLC16A5	hyper
SLC17A2	hyper
SLC17A6	hyper
SLC17A7	hyper
SLC19A1	hypo
SLC19A3	hyper
SLC1A1	hyper
SLC1A2	hyper
SLC1A4	hyper
SLC20A2	hyper
SLC22A16	hyper
SLC22A18	hyper
SLC22A2	hyper
SLC22A23	hyper
SLC22A3	hypo
SLC23A1	hyper
SLC23A3	hypo
SLC24A3	hyper
SLC24A4	hypo
SLC24A6	hypo
SLC25A1	hypo
SLC25A10	hyper
SLC25A13	hyper
SLC25A18	hyper
SLC25A24	hyper
SLC25A26	hypo
SLC25A3	hyper
SLC25A31	hyper
SLC25A33	hypo
SLC25A37	hypo
SLC25A40	hypo
SLC25A44	hypo

SLC25A46	hypo
SLC26A10	hyper
SLC26A5	hyper
SLC26A6	hypo
SLC26A9	hypo
SLC29A1	hypo
SLC29A3	hyper
SLC2A1	hyper
SLC2A13	hyper
SLC2A2	hyper
SLC2A5	hypo
SLC33A1	hypo
SLC34A1	hypo
SLC35A3	hyper
SLC35B2	hypo
SLC35B3	hyper
SLC35C2	hyper
SLC35D3	hypo
SLC35F1	hyper
SLC37A2	hypo
SLC37A3	hypo
SLC38A1	hypo
SLC39A1	hypo
SLC39A10	hypo
SLC39A14	hypo
SLC39A7	hypo
SLC39A8	hypo
SLC3A2	hyper
SLC43A2	hyper
SLC44A2	hypo
SLC44A4	hypo
SLC45A1	hypo
SLC45A3	hyper
SLC47A1	hyper
SLC4A4	hyper
SLC4A8	hypo
SLC5A1	hypo
SLC5A12	hypo
SLC5A7	hypo
SLC5A9	hypo
SLC6A1	hypo
SLC6A11	hyper
SLC6A12	hyper
SLC6A13	hyper
SLC6A15	hyper
SLC6A16	hypo
SLC6A18	hyper
SLC6A19	hypo
SLC6A20	hypo
SLC6A5	hyper
SLC6A6	hypo
SLC6A9	hyper
SLC7A11	hypo
SLC7A2	hyper
SLC7A4	hyper
SLC9A2	hyper
SLC9A9	hyper
SLFN13	hyper

SLFNL1	hypo
SLIT2	hyper
SLIT3	hypo
SLITRK1	hyper
SLMO2	hyper
SMAD3	hypo
SMAD5	hyper
SMAD6	hyper
SMAGP	hyper
SMARCA2	hypo
SMARCAD1	hyper
SMARCD1	hypo
SMARCD3	hypo
SMCP	hypo
SMPD3	hyper
SMPDL3A	hyper
SMURF2	hyper
SNAI1	hyper
SNAP91	hyper
SNAPC1	hypo
SNAPC2	hyper
SNAPC3	hypo
SNAR-I	hyper
SNCB	hypo
SNHG4	hypo
SNN	hyper
SNORA26	hypo
SNORA38	hypo
SNORA9	hypo
SNORD114-23	hypo
SNORD115-9	hyper
SNORD116-22	hyper
SNORD18C	hypo
SNORD1C	hypo
SNORD36C	hypo
SNORD45C	hypo
SNORD46	hypo
SNORD50B	hyper
SNORD9	hyper
SNPH	hypo
SNRK	hypo
SNRNP200	hyper
SNRNP35	hypo
SNRPA	hyper
SNTA1	hyper
SNTB1	hypo
SNTB2	hyper
SNTG1	hyper
SNTG2	hyper
SNUPN	hyper
SNX10	hypo
SNX11	hyper
SNX2	hyper
SNX20	hypo
SNX27	hypo
SNX29	hyper
SNX32	hyper
SNX4	hyper

SOBP	hyper
SOCS5	hyper
SOCS6	hyper
SOD1	hyper
SOD3	hyper
SOLH	hyper
SORCS1	hypo
SORCS3	hyper
SOS2	hypo
SOX11	hyper
SOX14	hyper
SOX21	hyper
SOX5	hypo
SOX8	hyper
SOX9	hyper
SP140L	hypo
SP7	hyper
SPACA3	hyper
SPAG4	hypo
SPAG6	hyper
SPAG7	hypo
SPATA18	hyper
SPATA2	hypo
SPATA20	hypo
SPATA21	hyper
SPATA22	hyper
SPATA5	hypo
SPATA7	hypo
SPATS1	hyper
SPDYA	hyper
SPEF1	hyper
SPEG	hyper
SPEN	hypo
SPHK2	hyper
SPHKAP	hyper
SPIRE2	hypo
SPNS2	hyper
SPO11	hypo
SPOCD1	hypo
SPOCK1	hypo
SPON1	hyper
SPOP	hyper
SPPL2B	hyper
SPRR2F	hypo
SPRY1	hyper
SPRY4	hyper
SPRYD3	hyper
SPSB1	hyper
SPSB4	hyper
SPTBN1	hyper
SQRDL	hypo
SR140	hypo
SRCIN1	hyper
SRD5A2	hyper
SRGAP1	hyper
SRGAP3	hyper
SRI	hyper
SRMS	hypo

SRP9	hypo
SRPRB	hyper
SRRM1	hypo
SRXN1	hypo
SS18	hypo
SSBP3	hyper
SSFA2	hyper
SSR1	hypo
SSRP1	hyper
SST	hyper
SSU72	hyper
ST18	hyper
ST3GAL1	hypo
ST3GAL2	hypo
ST5	hyper
ST6GAL2	hyper
ST6GALNAC1	hypo
ST6GALNAC4	hypo
ST6GALNAC5	hypo
ST6GALNAC6	hyper
ST7OT3	hyper
ST8SIA1	hyper
ST8SIA3	hyper
ST8SIA5	hyper
STAC2	hyper
STAG3	hyper
STARD13	hyper
STARD9	hypo
STAT1	hypo
STAT2	hypo
STK19	hyper
STK24	hyper
STMN1	hypo
STMN3	hyper
STOM	hyper
STOX2	hypo
STRADA	hypo
STRBP	hypo
STRN	hyper
STX16	hypo
STX18	hyper
STX4	hypo
STXBP5L	hyper
STYXL1	hypo
SUGT1L1	hypo
SULF2	hyper
SUSD5	hypo
SUV420H1	hyper
SV2C	hyper
SVIL	hypo
SYCP1	hyper
SYF2	hyper
SYNE1	hyper
SYNGR3	hyper
SYNJ2	hyper
SYNPR	hyper
SYT14	hyper
SYT5	hyper

SYT7	hyper
SYT9	hyper
SYTL3	hypo
T	hyper
TAAR2	hyper
TAC1	hypo
TAC3	hypo
TACO1	hypo
TAF12	hypo
TAF1B	hypo
TAF1D	hypo
TAF4B	hypo
TAGLN3	hypo
TANC1	hypo
TANC2	hyper
TAOK3	hypo
TAPBP	hyper
TAPBPL	hypo
TARS2	hypo
TARSL2	hyper
TAS1R1	hyper
TAS1R2	hyper
TAS2R40	hypo
TBC1D1	hyper
TBC1D10A	hypo
TBC1D15	hypo
TBC1D16	hypo
TBC1D17	hypo
TBC1D19	hypo
TBC1D2	hypo
TBC1D22A	hyper
TBC1D5	hypo
TBC1D8	hypo
TBCA	hypo
TBCC	hyper
TBL1XR1	hyper
TBL2	hypo
TBRG4	hypo
TBX15	hyper
TBX2	hyper
TBX20	hyper
TBX3	hyper
TBX4	hyper
TBX5	hyper
TBXA2R	hyper
TC2N	hypo
TCEA2	hyper
TCEA3	hyper
TCEB1	hypo
TCF12	hypo
TCF19	hypo
TCF21	hyper
TCF25	hyper
TCF3	hyper
TCF4	hyper
TCF7L1	hypo
TCF7L2	hyper
TCFL5	hyper

TCHHL1	hyper
TCIRG1	hyper
TCL1A	hypo
TCL6	hypo
TCP11L2	hypo
TCTE1	hypo
TCTEX1D1	hyper
TCTEX1D4	hypo
TDH	hyper
TDRD10	hypo
TEAD1	hyper
TEAD4	hyper
TECR	hypo
TENC1	hyper
TET1	hyper
TEX14	hyper
TFAP2D	hyper
TFAP4	hyper
TFDP1	hypo
TFF2	hypo
TFG	hypo
TFPI2	hyper
TGFB1I1	hyper
TGFBI	hyper
TGIF1	hypo
TGM2	hyper
TGM3	hypo
TGOLN2	hypo
THAP9	hypo
THBD	hyper
THBS4	hyper
THG1L	hyper
THOC1	hypo
THRB	hyper
THRSP	hypo
THSD1P	hypo
THSD4	hypo
TIAL1	hypo
TIAM2	hypo
TIMM22	hypo
TIMM44	hypo
TIMP2	hypo
TINAGL1	hyper
TIRAP	hypo
TKTL2	hypo
TLE2	hypo
TLE3	hypo
TLN2	hyper
TLR9	hypo
TLX1	hyper
TLX1NB	hyper
TLX3	hyper
TM4SF19	hypo
TM9SF1	hyper
TM9SF2	hypo
TMC1	hyper
TMC2	hypo
TMC4	hyper

TMC5	hyper
TMCO2	hyper
TMCO3	hyper
TMCO6	hypo
TMED7-TICAM2	hypo
TMED9	hyper
TMEM100	hyper
TMEM120A	hypo
TMEM126A	hyper
TMEM130	hyper
TMEM131	hyper
TMEM132E	hyper
TMEM161B	hypo
TMEM171	hyper
TMEM174	hyper
TMEM175	hyper
TMEM176B	hyper
TMEM178	hyper
TMEM179	hyper
TMEM18	hypo
TMEM185B	hyper
TMEM186	hypo
TMEM194B	hypo
TMEM195	hyper
TMEM2	hyper
TMEM200B	hyper
TMEM200C	hypo
TMEM206	hypo
TMEM208	hypo
TMEM209	hyper
TMEM215	hypo
TMEM220	hyper
TMEM222	hyper
TMEM229A	hyper
TMEM45B	hyper
TMEM51	hyper
TMEM55A	hypo
TMEM56	hypo
TMEM62	hypo
TMEM82	hyper
TMEM88B	hypo
TMEM8B	hyper
TMEM97	hypo
TMIGD2	hypo
TMOD4	hyper
TMPRSS2	hyper
TMPRSS3	hypo
TMPRSS5	hyper
TMPRSS6	hypo
TMTC4	hyper
TNFAIP2	hyper
TNFAIP8L1	hypo
TNFRSF11B	hyper
TNFRSF13B	hypo
TNFRSF18	hypo
TNFRSF1A	hypo
TNFSF13	hypo
TNKS	hyper

TNNC1	hypo
TNNT2	hypo
TNRC6B	hypo
TNRC6C	hyper
TOLLIP	hyper
TOMM40	hypo
TOMM5	hypo
TOMM7	hyper
TOP2A	hypo
TOR3A	hyper
TOX	hyper
TOX3	hyper
TP53AIP1	hypo
TP53BP1	hyper
TP53I13	hypo
TP53I3	hyper
TP73	hypo
TPBG	hyper
TPI1	hypo
TPK1	hyper
TPM1	hyper
TPO	hyper
TPRG1L	hyper
TPST1	hyper
TPST2	hypo
TPX2	hypo
TRA2B	hyper
TRADD	hyper
TRAF1	hyper
TRAF2	hyper
TRAF7	hyper
TRAM2	hyper
TRAPPC6B	hyper
TRDN	hyper
TRHDE	hyper
TRIM10	hypo
TRIM11	hypo
TRIM2	hyper
TRIM27	hyper
TRIM34	hypo
TRIM36	hyper
TRIM37	hypo
TRIM38	hypo
TRIM41	hypo
TRIM42	hyper
TRIM44	hyper
TRIM47	hypo
TRIM5	hypo
TRIM58	hyper
TRIM67	hypo
TRIM6-TRIM34	hypo
TRIM72	hypo
TRIP12	hypo
TRIP4	hyper
TRMT11	hypo
TRPC6	hyper
TRPM2	hyper
TRPM5	hypo

TRPV1	hypo
TRPV4	hyper
TRRAP	hyper
TRUB2	hyper
TSC22D1	hypo
TSGA10	hypo
TSHZ2	hyper
TSN	hyper
TSNAXIP1	hypo
TSPAN13	hyper
TSPAN17	hypo
TSPAN4	hypo
TSPAN9	hyper
TSPO	hyper
TSSC1	hyper
TTBK1	hypo
TTC24	hypo
TTC39A	hyper
TTC9B	hyper
TTK	hyper
TTLL13	hyper
TTLL8	hyper
TTYH2	hypo
TUB	hypo
TUBB	hypo
TUBB2B	hypo
TUBG2	hyper
TUBGCP6	hyper
TUFT1	hyper
TWIST1	hyper
TWISTNB	hypo
TWSG1	hypo
TXN2	hyper
TXNDC11	hypo
TXNDC17	hypo
TXNDC9	hypo
TXNL4B	hypo
TXNRD1	hypo
TYMS	hyper
TYR	hypo
UBAC2	hypo
UBD	hypo
UBE2B	hypo
UBE2CBP	hypo
UBE2D1	hyper
UBE2D3	hypo
UBE2I	hyper
UBE2K	hyper
UBE2L3	hyper
UBE2N	hypo
UBE2Q1	hyper
UBE2S	hypo
UBE2Z	hypo
UBE3C	hypo
UBFD1	hypo
UBIAD1	hypo
UBL3	hypo
UBL4B	hypo

UBL7	hypo
UBP1	hyper
UBR1	hypo
UBR3	hypo
UBR5	hyper
UBTD2	hypo
UBTF	hyper
UBXN1	hyper
UBXN10	hypo
UCK2	hyper
UCKL1	hyper
UCN3	hyper
UCP1	hyper
UFSP2	hypo
UGCG	hypo
UGDH	hypo
UGP2	hypo
UGT1A10	hyper
UGT3A1	hyper
UGT8	hyper
UIMC1	hyper
ULK1	hypo
UNC5A	hyper
UNC5C	hyper
UNC5D	hypo
UNC80	hyper
UNC84A	hypo
UNCX	hyper
UNKL	hypo
UPF1	hypo
UPK1A	hyper
UPK3A	hyper
UPP2	hypo
URM1	hypo
USF1	hyper
USH1G	hyper
USH2A	hyper
USO1	hyper
USP10	hyper
USP13	hyper
USP14	hyper
USP15	hypo
USP18	hypo
USP2	hyper
USP24	hyper
USP28	hypo
USP29	hyper
USP4	hypo
USP40	hypo
USP45	hyper
USP46	hypo
USP49	hyper
USP5	hypo
USP50	hyper
USP6NL	hypo
USP7	hypo
USPL1	hypo
UST	hypo

UTRN	hyper
UTS2R	hyper
UVRAG	hypo
VAMP8	hyper
VARS	hyper
VARS2	hyper
VASH1	hyper
VAT1L	hypo
VAX1	hyper
VCAN	hypo
VEGFA	hypo
VENTX	hyper
VEPH1	hyper
VIM	hypo
VIPR1	hyper
VIPR2	hypo
VIT	hypo
VNN1	hyper
VOPP1	hyper
VPS13D	hyper
VPS52	hyper
VPS72	hypo
VPS8	hyper
VRK3	hypo
VSIG10	hypo
VSTM2A	hyper
VSTM2B	hyper
VSTM2L	hyper
VSX2	hyper
VTRNA1-3	hyper
VWA5A	hypo
VWA5B1	hyper
VWC2	hypo
WASF3	hypo
WBP1	hypo
WBP2NL	hyper
WBSCR16	hypo
WBSCR17	hyper
WDFY1	hyper
WDFY2	hypo
WDR1	hyper
WDR18	hyper
WDR20	hypo
WDR26	hyper
WDR33	hyper
WDR37	hyper
WDR5	hyper
WDR51A	hypo
WDR52	hyper
WDR60	hyper
WDR64	hyper
WDR66	hyper
WDR69	hyper
WDR70	hypo
WDR75	hyper
WDR8	hypo
WDR81	hyper
WDR90	hypo

WEE2	hypo
WFDC10B	hypo
WFS1	hyper
WIPF3	hyper
WIPI1	hypo
WIPI2	hyper
WNT1	hypo
WNT10A	hypo
WNT10B	hyper
WNT2	hyper
WNT2B	hyper
WNT3A	hyper
WNT5B	hypo
WNT6	hypo
WNT7B	hyper
WNT9A	hyper
WSB1	hypo
WSCD1	hypo
WWP2	hyper
WWTR1	hypo
XIRP1	hypo
XKR6	hyper
XPNPEP3	hypo
XPO4	hyper
XPO6	hypo
XPO7	hyper
XRN2	hypo
YARS	hypo
YIF1A	hypo
YIPF4	hypo
YJEFN3	hyper
YOD1	hyper
YPEL1	hyper
YPEL3	hyper
YPEL4	hyper
YTHDF2	hypo
YWHAG	hyper
ZAK	hyper
ZAP70	hyper
ZBED2	hypo
ZBTB17	hypo
ZBTB2	hypo
ZBTB20	hypo
ZBTB22	hyper
ZBTB34	hypo
ZBTB39	hyper
ZBTB44	hypo
ZBTB46	hypo
ZBTB47	hyper
ZBTB6	hypo
ZBTB7A	hypo
ZBTB8B	hyper
ZC3H10	hypo
ZC3H12A	hyper
ZC3H13	hyper
ZC3H14	hypo
ZC3H18	hyper
ZC3H3	hyper

ZC3H4	hyper
ZC3H7A	hyper
ZCCHC2	hypo
ZCCHC7	hyper
ZDHHC17	hyper
ZDHHC20	hypo
ZDHHC21	hyper
ZDHHC7	hyper
ZEB1	hyper
ZFAND3	hypo
ZFAT	hyper
ZFP161	hypo
ZFP82	hyper
ZFPM1	hyper
ZFR2	hypo
ZFYVE16	hypo
ZFYVE21	hyper
ZIC1	hyper
ZIC4	hyper
ZIK1	hyper
ZIM2	hyper
ZKSCAN5	hyper
ZMYND11	hyper
ZNF114	hyper
ZNF12	hypo
ZNF132	hyper
ZNF140	hypo
ZNF141	hypo
ZNF146	hypo
ZNF167	hyper
ZNF17	hyper
ZNF197	hyper
ZNF200	hyper
ZNF202	hyper
ZNF212	hypo
ZNF213	hypo
ZNF214	hyper
ZNF215	hyper
ZNF221	hypo
ZNF23	hyper
ZNF232	hyper
ZNF239	hypo
ZNF256	hyper
ZNF283	hyper
ZNF296	hyper
ZNF302	hyper
ZNF311	hyper
ZNF318	hyper
ZNF324B	hyper
ZNF331	hyper
ZNF34	hypo
ZNF341	hypo
ZNF354A	hyper
ZNF354C	hypo
ZNF37A	hyper
ZNF384	hyper
ZNF395	hyper
ZNF398	hyper

ZNF415	hyper
ZNF423	hyper
ZNF425	hypo
ZNF428	hypo
ZNF43	hypo
ZNF44	hypo
ZNF444	hyper
ZNF445	hypo
ZNF454	hyper
ZNF467	hyper
ZNF469	hyper
ZNF501	hyper
ZNF507	hypo
ZNF512	hypo
ZNF517	hyper
ZNF521	hypo
ZNF529	hyper
ZNF540	hyper
ZNF544	hyper
ZNF554	hyper
ZNF557	hypo
ZNF558	hypo
ZNF559	hyper
ZNF563	hypo
ZNF57	hypo
ZNF572	hypo
ZNF574	hyper
ZNF581	hypo
ZNF582	hyper
ZNF595	hyper
ZNF599	hyper
ZNF605	hyper
ZNF607	hyper
ZNF609	hyper
ZNF610	hyper
ZNF616	hyper
ZNF618	hypo
ZNF620	hyper
ZNF622	hypo
ZNF623	hyper
ZNF646	hypo
ZNF665	hyper
ZNF667	hyper
ZNF668	hyper
ZNF670	hypo
ZNF687	hyper
ZNF688	hypo
ZNF702P	hyper
ZNF709	hyper
ZNF71	hypo
ZNF710	hyper
ZNF718	hyper
ZNF74	hypo
ZNF763	hyper
ZNF773	hypo
ZNF774	hypo
ZNF775	hypo
ZNF776	hyper

ZNF787	hyper
ZNF792	hypo
ZNF793	hyper
ZNF8	hypo
ZNF80	hyper
ZNF816A	hyper
ZNF823	hyper
ZNF827	hyper
ZNF830	hypo
ZNF831	hypo
ZNF835	hyper
ZNF837	hyper
ZNF841	hyper
ZNF844	hyper
ZNF860	hypo
ZNF878	hyper
ZNF92	hyper
ZNHIT2	hypo
ZNRF2	hypo
ZNRF3	hypo
ZSCAN18	hyper
ZSCAN2	hypo
ZSWIM6	hypo
ZSWIM7	hypo
ZXDC	hypo
ZYG11A	hyper
ZYX	hyper
